# Theoretical foundations of soft topological granular structures for multi-criteria social network analysis

**DOI:** 10.1098/rsos.260366

**Published:** 2026-09-09

**Authors:** Amlanjyoti Oza

**Affiliations:** ^1^ Department of Mathematics, Jawaharlal Nehru College, Boko, Kamrup, Assam, India

**Keywords:** soft topology, granular computing, multi-criteria social network analysis

## Abstract

Granular computing (GrC) models complex systems through hierarchically organized information granules, whereas soft set theory offers a flexible mathematical framework for parameter-dependent uncertainty. Motivated by their strong conceptual affinity, this paper develops a soft topological GrC framework grounded in Molodtsov's proximity-oriented soft topology, which is unexplored to a great extent until now by the researchers of soft computing and related areas. New granular operators, reachability relations and saturation concepts are introduced, which lead to a unified structure that integrates soft neighbourhoods with multi-level granularity. Several non-trivial theorems are established concerning granular closure, connectivity, convergence and structural equivalence with examples, which reveals the intrinsic multi-resolution nature of soft topological spaces. The theoretical framework is further interpreted in the context of multi-criteria social network analysis, where contextual interactions induce soft neighbourhoods, and granular communities represent stable influence formations. The present study is primarily theoretical in nature and lays a rigorous foundation for future computational and empirical investigations.

## 1. Introduction

The analysis of complex systems often requires mathematical tools capable of handling uncertainty, context dependence and multi-level structural organization. Classical models based on crisp relations and binary membership [1] are frequently inadequate for capturing the nuanced and parameter-dependent interactions that arise in real-world systems such as social networks [2], decision making environments [3] and information systems [4]. This limitation has led to the development of several complementary theories, including fuzzy sets [5], rough sets [6], soft sets [7], granular computing (GrC) [8], etc.

Soft set theory, introduced by Molodtsov [7], has evolved into a rich framework for uncertainty modelling and has generated extensive developments in soft topology [9–11], decision analysis [12,13], information systems [14,15], etc. Since its introduction by Molodtsov [7], soft set theory has undergone extensive generalizations and has evolved into a rich family of uncertainty models. Among these developments, fuzzy soft sets and their recent generalizations, such as (a,b)-fuzzy soft sets, have been proposed to provide greater flexibility in representing graded uncertainty and parametrized vagueness [16,17]. Neutrosophic soft sets have further extended the framework by incorporating indeterminacy and have found applications in multi-criteria decision-making environments [18]. Similarly, bipolar soft sets and their variants have been developed to simultaneously represent positive and negative information and have led to several new decision-making methodologies and uncertainty models [19,20]. More recently, hypersoft sets and advanced extensions such as superhypersoft sets and related higher-order structures have been introduced to model complex multi-attribute interactions and hierarchical parameter dependencies [21,22]. Recent years also have witnessed increasing interest in hybrid uncertainty frameworks that integrate soft computing methodologies with granular representations [23], intelligent decision systems [12,13] and network-oriented models [24]. Several recent studies have also explored topological structures [9–11] derived from enriched soft set models for relationship analysis and decision-support environments. For example, topological frameworks based on virtual fuzzy parametrized soft sets have been employed to enhance the analysis of relational structures and uncertain interactions [25]. These developments demonstrate the effectiveness of combining parametrized uncertainty models, topological structures and relational reasoning mechanisms in complex environments. However, most of these studies focus on enriched soft set variants, decision-support mechanisms or application specific constructions. Nevertheless, despite the success of these enriched frameworks, the foundational interaction between the original proximity-based soft topology and GrC remains comparatively underexplored from a rigorous mathematical perspective.

For example, much of the existing literature has been oriented towards applications [26–28], hybrid models [29,30] and simulation-based studies [31,32], often leaving its foundational and structural aspects comparatively underexplored. Also, soft topological structures have primarily concentrated on extending topological concepts to enriched soft set models, establishing separation axioms [33,34], continuity [35], compactness [36] and decision-support methodologies under various uncertainty paradigms. On the other hand, GrC research has largely focused on information granules constructed from equivalence relations [1,37], rough sets [38–41] and fuzzy models [42]. Although, these developments have significantly advanced their respective fields, the original formulation of soft set theory provides a mathematically rigorous and conceptually flexible framework for modelling uncertainty driven situations without imposing probabilistic, fuzzy or metric assumptions. In particular, the soft topological structures introduced by Molodtsov [43] offer a rich foundation for analysing neighbourhood formation, connectivity and convergence under parameter dependence. Motivated by this observation, the present work deliberately departs from simulation-centric or experimental investigations and instead focuses on the development of a foundational GrC framework grounded in soft topological spaces. By restricting attention to the underlying theory, we aim to clarify the structural mechanisms through which stable granules, communities and influence domains emerge in multi-criteria environments, thereby strengthening the theoretical core upon which future algorithmic and empirical studies can be systematically built. On the other hand, GrC has emerged as a unifying paradigm for representing and processing information through granules: collections of objects grouped by similarity, functionality or indistinguishability [8,37,44,45]. Notions, such as hierarchy [46], multi-resolution modelling [47] and adaptive information processing [47], are central to GrC, which enable analysis at different levels of abstraction. It has evolved into a well-established paradigm for information processing through the construction, manipulation and analysis of information granules. Since its formalization, GrC has been extensively integrated with rough sets [38–41], fuzzy sets [42], neighbourhood systems [48] and topology [49] to address uncertainty, hierarchical knowledge representation and multi-level reasoning. Recent studies have further expanded the scope of GrC by developing hybrid granular models that combine soft sets with fuzzy sets [50], intuitionistic fuzzy sets [51], neutrosophic soft sets [52] and other uncertainty frameworks, leading to significant advances in decision-making, pattern recognition, knowledge discovery and intelligent information systems. Despite these developments, the construction of granular structures directly from Molodtsov's original proximity-based soft topology has received very limited attention. In particular, the theoretical relationship between proximity-induced soft neighbourhood systems and granular structures has not been systematically investigated. Motivated by this research gap, the present work establishes a unified soft topological granular framework that derives information granules from proximity-based soft topological neighbourhoods. New notions such as granular closure, saturation, reachability and equivalence are defined and analysed. We establish several mathematically rigorous results that characterize the behaviour of these structures, including existence, convergence and connectivity theorems. These results provide a theoretical foundation for hierarchical and multi-resolution reasoning within soft topological environments. For more intuition, we present the correspondence between soft topology and GrC in table 1.

**Table 1.** Correspondence between soft topology and GrC.

soft topological concept	GrC interpretation
τ(x)	local information granule centred at x
$G_{\tau }=\{\tau (x):x\in X\}$	soft topological granular structure (family of information granules)
overlapping τ-neighbourhoods	shared contexts and multi-criteria interactions among elements
iterated neighbourhood expansion	granular influence propagation process
granular closure ${\overset{―}{A}}_{g}^{\;\tau }$	stabilized region of influence generated by a set A
granular community C	cohesive information cluster induced by local neighbourhoods
stable core of a granular community	structurally robust agents whose neighbourhoods remain inside the community
directed systems of granular structures	hierarchical and multi-resolution representations of information granules

Thus, the main theoretical contributions of this paper are summarized as follows:

— Introduction of the notion of a soft topological granular structure based on proximity-induced soft neighbourhoods.
— Development of granular closure, granular saturation and reachability operators within soft topological environments.
— Establishment of structural properties concerning connectivity, convergence and stability of granular systems.
— Characterization of granular equivalence relations and invariant granular regions.

To illustrate the interpretative potential of the proposed framework, we discuss its applicability to multi-criteria social network analysis with some mathematical theorems and illustrative examples.

Modern social systems are inherently multi-criteria in nature, with relationships among individuals arising simultaneously from several factors such as friendship [53], professional collaboration [54], trust [55,56], communication frequency [24,53], shared interests [24,53] and common activities [24,53]. Classical social network analysis models typically represent networks using a single type of relation or a weighted aggregation of multiple relations. Although such approaches have been highly successful in studying network structure and dynamics, they often fail to adequately capture the context-dependent and overlapping nature of social interactions.

To address these limitations, several frameworks for multi-criteria social network analysis have been developed, including multiplex networks [57], multi-layer networks [58] and attributed social networks [59]. These models permit the coexistence of multiple relation types and provide richer representations of complex social systems. However, many existing approaches remain primarily graph-theoretic in nature and generally rely on fixed relational structures. Consequently, they may not fully account for the local and parameter-dependent organization of information, where an individual can simultaneously participate in multiple contexts and where communities often exhibit overlapping and hierarchical structures.

From the perspective of GrC, information is processed and reasoned about through collections of locally coherent objects called information granules. The local and overlapping nature of soft topological neighbourhoods therefore suggests a natural granular interpretation. By viewing each τ-neighbourhood as an information granule and the collection of all such neighbourhoods as a granular system, one obtains a flexible framework capable of describing influence propagation, community formation and hierarchical organization in multi-criteria social networks. This observation serves as one of the principal motivations of the present work and provides the conceptual basis for the soft topological granular structures developed in the subsequent sections.

In particular, the soft topological granular model is employed to address the following fundamental questions:

— How do contextual and multi-criteria interactions induce stable granular communities in a social network?
— Under what conditions does influence propagate and converge within such granular structures?
— Which agents form structurally influential cores whose induced neighbourhoods remain invariant under multi-criteria influence propagation?

The paper is organized as follows. Section 2 recalls the necessary foundations of soft set theory, soft topology and GrC. In §3, we develop the soft topological granular framework and establish its fundamental properties with some suitable examples. Section 4 presents applications to multi-criteria social network analysis with a synthetic network as an example for illustration of the theoretical developments. Finally, §5 concludes the paper with a discussion of potential extensions and future research directions.

## 2. Foundations of soft set theory and granular computing

Soft set theory [7,43,60,61] and GrC [8,45,46,62] provide complementary mathematical foundations for modelling uncertainty and hierarchical information structures. Below, we summarize the core notions required for the subsequent development.

### 2.1. Soft set theory and soft topological spaces

The mathematical treatment of uncertainty, vagueness and parameter-dependent information has been shaped by several complementary frameworks [5,7,8,63,64], among which soft set theory is a fundamental approach. The mathematical definition of a soft set is given below.

**Definition 2.1.**

[7] A soft set over the universal set X is a pair (S,A) where S is a mapping from the set of parameters A into the set of all subsets of X, i.e. $S:A\to 2^{X}$. In fact, a soft set is a parametrized family of subsets. For a given soft set (S,A), the family τ(S,A) can be defined as τ(S,A)={S(a):a∈A}.

In [61], Molodtsov wrote regarding soft set theory and soft topology as: ‘To determine a topology, we have to define only the family of vicinities of a point. No comparison of vicinities and no other properties of these subsets are needed. The situation is quite similar for soft sets, as a soft set is a family of vicinities of a point, except that the initial point (as in topology) may not exist. Thus, the role of parameters in the definition of soft sets is only auxiliary. Parameters are used only as names of subsets. Therefore, the introduction of the notion of equivalence of soft sets (S,A) and $\left(S^{\prime },A^{\prime }\right)$ should be based on the equality of families of sets τ(S,A) and $\tau (S^{\prime },A^{\prime })$, but not on the equality of point to set mappings S and $S^{\prime }$’.

Thus, in this work, we adopt the original formulations of soft set theory and soft topology as introduced by Molodtsov, the founder of soft set theory. Consequently, our focus is restricted to these classical formulations, and alternative extensions and modifications proposed by other authors, though widely applied across various domains, are beyond the scope of the present study.

**Definition 2.2.**

[60] Two soft sets (S,A) and $\left(S^{\prime },A^{\prime }\right)$ given over the universal set X will be called equal, and we write $\left(S,A)=(S^{\prime },A^{\prime }\right)$ if and only if $S=S^{\prime }$ and $A=A^{\prime }$.

**Definition 2.3.**

[60] Two soft sets (S,A) and $\left(S^{\prime },A^{\prime }\right)$ given over the universal set X will be called equivalent and written as $\left(S,A)≅(S^{\prime },A^{\prime }\right)$ if and only if $\tau (S,A)=\tau (S^{\prime },A^{\prime })$.

Here, we provide some basic definitions regarding soft set theory. For a more detailed discussion, see [7,43,60,61].

After recalling these basic notions of soft set theory, it is natural to include the topological structure [43] that captures parameter-dependent notions of neighbourhoods, openness, connectedness, compactness, etc. A soft topological space extends the classical concept of topology by replacing crisp open neighbourhoods with a soft set indexed by a set of parameters. This allows for the description of local properties of elements relative to different contextual viewpoints, which is particularly suitable for modelling uncertainty and multi-criteria information. These structures provide the mathematical foundation for defining neighbourhood systems and discussions of soft environments and play a central role in the development of soft granular topological frameworks considered in this work. Here, we include only those definitions and notions of soft topological spaces that are necessary for this paper.

**Definition 2.4.**

[43] Let X be a non-empty set. A mapping $\tau :X\to 2^{X}$ is called a proximity mapping if x∈τ(x),∀x∈X. The set τ(x) is called the τ-neighbourhood of the point x. We call (X,τ) the soft topological space on X endowed with a proximity mapping τ.

**Definition 2.5.**

[43] Let (X,τ) be a soft topological space on X endowed with a proximity mapping τ. For a non-empty subset A⊆X, the τ-neighbourhood of A is defined as

$$\text{Close}(A,X,\tau )=\underset{x\in A}{⋃}\tau (x).$$

We additionally assume that Close(∅,X,τ)=∅.

**Definition 2.6.**

[43] Let (X,τ) be a soft topological space on X endowed with a proximity mapping τ. The set of τ-interior points of A is defined by

Int(A,X,τ)={x∈X:τ(x)⊆A}.

**Definition 2.7.**

[43] Let (X,τ) be a soft topological space on X endowed with a proximity mapping τ. The τ-boundary of A is defined as

Bord(A,X,τ)=Close(A,X,τ)∩Close(X∖A,X,τ).

**Definition 2.8.**

[43] Let (X,τ) be a soft topological space on X endowed with a proximity mapping τ. The set of τ-hidden points of A is defined by

Bow(A,X,τ)=A∖Bord(A,X,τ).

**Definition 2.9.**

[43] Let (X,τ) be a soft topological space on X endowed with a proximity mapping τ and let A,B⊆X. The sets A and B are said to be τ-separable if at least one of the following conditions holds:

Close(A,X,τ)∩B=∅,A∩Close(B,X,τ)=∅.

**Definition 2.10.**

[43] Let (X,τ) be a soft topological space on X endowed with a proximity mapping τ and let A,B⊆X. The sets A and B are called strongly τ-separable if both of the following conditions are satisfied:

Close(A,X,τ)∩B=∅,A∩Close(B,X,τ)=∅.

**Definition 2.11.**

[43] Let (X,τ) be a soft topological space on X endowed with a proximity mapping τ. A finite sequence of points $\left(x_{1},x_{2},\ldots ,x_{n}\right)$ with $x_{i}\in X$, and n≥1 is called a τ-path if

$$x_{i+1}\in \tau (x_{i}),\text{for all }i=1,2,\ldots ,n-1.$$

Paths consisting of a single point are permitted; in this case, the point serves as both the initial and terminal point of the path. If a,b∈X, the collection of all τ-paths with initial point a and terminal point b is denoted by Path(X,τ,a,b).

**Definition 2.12.**

[43] Let $\lambda =(x_{1},\ldots ,x_{n})\in \text{Path}(X,\tau ,a,b)$ and $\mu =(y_{1},\ldots ,y_{m})\in \text{Path}(X,\tau ,c,d)$. The concatenation of λ and μ is defined as

$$\lambda *\mu =(x_{1},\ldots ,x_{n},y_{1},\ldots ,y_{m}),$$

provided that c∈τ(b).

**Definition 2.13.**

[43] Let (X,τ) be a soft topological space on X endowed with a proximity mapping τ and let A⊆X. For a,b∈A, the set of all τ-paths from a to b consisting entirely of points in A is denoted by ${\text{Path}}_{A}(X,\tau ,a,b).$

**Definition 2.14.**

[43] Let (X,τ) be a soft topological space on X endowed with a proximity mapping τ, let A⊆X and let a∈A. The τ-reachability set of a in A is defined as

$${\text{Reach}}_{A}(X,\tau ,a)=\{b\in A:{\text{Path}}_{A}(X,\tau ,a,b)\neq \emptyset \}.$$

**Definition 2.15.**

[43] Let (X,τ) be a soft topological space on X endowed with a proximity mapping τ and let A⊆X. Then the communication nodes (or hubs) of A are defined as

$$\text{Hub}(X,A,\tau )=\{a\in A:{\text{Reach}}_{A}(X,\tau ,a)=A\}.$$

**Definition 2.16.**

[43] Let (X,τ) be a soft topological space on X endowed with a proximity mapping τ and let A⊆X.

— A is called τ-semi connected if Hub(X,A,τ)≠∅.
— A is called τ-connected if Hub(X,A,τ)=A.

Now, consider the following example, which illustrates the above-mentioned definitions in a unified setting.

**Example 2.1.**

Let $X=\{x_{1},x_{2},x_{3},x_{4},x_{5}\}$. Let the parameter set be $A=\{a_{1},a_{2},a_{3}\}$. Define a soft set (S,A) over X by $S(a_{1})=\{x_{1},x_{2},x_{3}\}$, $S(a_{2})=\{x_{2},x_{4}\}$ and $S(a_{3})=\{x_{1},x_{4},x_{5}\}$. Hence, $\tau (S,A)=\{\{x_{1},x_{2},x_{3}\},\{x_{2},x_{4}\},\{x_{1},x_{4},x_{5}\}\}$. Now, consider another parameter set $A^{\prime }=\{b_{1},b_{2},b_{3}\}$ and define the soft set $\left(S^{\prime },A^{\prime }\right)$ by $S^{\prime }(b_{1})=\{x_{2},x_{4}\}$, $S^{\prime }(b_{2})=\{x_{1},x_{4},x_{5}\}$ and $S^{\prime }(b_{3})=\{x_{1},x_{2},x_{3}\}$. Then, $\tau (S^{\prime },A^{\prime })=\{\{x_{1},x_{2},x_{3}\},\{x_{2},x_{4}\},\{x_{1},x_{4},x_{5}\}\}=\tau (S,A)$. Since $A\neq A^{\prime }$ and $S\neq S^{\prime }$, we have $\left(S,A)\neq (S^{\prime },A^{\prime }\right)$, but $\left(S,A)≅(S^{\prime },A^{\prime }\right)$.

Next, define a proximity mapping $\tau :X\to 2^{X}$ by $\tau (x_{1})=\{x_{1},x_{2}\},\tau (x_{2})=\{x_{2},x_{3}\},\tau (x_{3})=\{x_{3},x_{4}\},\tau (x_{4})=\{x_{4},x_{5}\},\text{and}\;\tau (x_{5})=\{x_{5}\}$.

Since $x_{i}\in \tau (x_{i})$ for every i, (X,τ) is a soft topological space. Let $C=\{x_{2},x_{3},x_{4}\}\subseteq X$. Then, $\text{Close}(C,X,\tau )=\tau (x_{2})\cup \tau (x_{3})\cup \tau (x_{4})=\{x_{2},x_{3},x_{4},x_{5}\}$. Also, $\text{Int}(C,X,\tau )=\{x\in X:\tau (x)\subseteq C\}=\{x_{2},x_{3}\}$, because $\tau (x_{2})=\{x_{2},x_{3}\}\subseteq C$ and $\tau (x_{3})=\{x_{3},x_{4}\}\subseteq C$ and no other τ-neighbourhood is contained in C. Since $X\setminus C=\{x_{1},x_{5}\}$, we obtain $\text{Close}(X\setminus C,X,\tau )=\tau (x_{1})\cup \tau (x_{5})=\{x_{1},x_{2},x_{5}\}$.

Therefore, $\text{Bord}(C,X,\tau )=\text{Close}(C,X,\tau )\cap \text{Close}(X\setminus C,X,\tau )=\{x_{2},x_{5}\}$. Hence, $\text{Bow}(C,X,\tau )=C\setminus \text{Bord}(C,X,\tau )=\{x_{3},x_{4}\}$.

Consider $P=\{x_{1}\},\;Q=\{x_{4}\}$. Then, $\text{Close}(P,X,\tau )=\{x_{1},x_{2}\}$, and therefore Close(P,X,τ)∩Q=∅. Also, $\text{Close}(Q,X,\tau )=\{x_{4},x_{5}\}$, and hence P∩Close(Q,X,τ)=∅. Therefore, P and Q are strongly τ-separable and consequently τ-separable.

A τ-path from $x_{1}$ to $x_{5}$ is $\lambda =(x_{1},x_{2},x_{3},x_{4},x_{5})$, because $x_{2}\in \tau (x_{1}),\;x_{3}\in \tau (x_{2}),\;x_{4}\in \tau (x_{3})\text{ and}\;x_{5}\in \tau (x_{4})$. Another path is $\mu =(x_{5})$. Since $x_{5}\in \tau (x_{5})$, the concatenation is defined and equals $\lambda *\mu br=(x_{1},x_{2},x_{3},x_{4},x_{5},x_{5})$.

Let $A_{1}=\{x_{1},x_{2},x_{3},x_{4},x_{5}\}=X$. Then ${\text{Path}}_{A_{1}}(X,\tau ,x_{1},x_{5})\neq \emptyset$ because the path λ lies entirely in $A_{1}$. Moreover, ${\text{Reach}}_{A_{1}}(X,\tau ,x_{1})=X$, since every element of X can be reached from $x_{1}$. Hence, $\text{Hub}(X,A_{1},\tau )=\{x_{1}\}$.

Therefore, $\text{Hub}(X,A_{1},\tau )\neq \emptyset$, so $A_{1}$ is τ-semi connected. However, $\text{Hub}(X,A_{1},\tau )\neq A_{1}$, and thus $A_{1}$ is not τ-connected.

### 2.2. Fundamentals of granular computing

GrC is a general computational and cognitive paradigm that focuses on the representation, processing and reasoning of information through information granules rather than individual objects [8,46,47]. An information granule is typically understood as a collection of entities grouped together owing to similarity, proximity, indistinguishability or functional coherence [8]. This paradigm reflects the way humans naturally perceive and analyse complex systems by shifting attention across multiple levels of abstraction [65].

From a mathematical viewpoint, GrC does not prescribe a single formal model. Instead, it provides a unifying framework under which various theories such as rough sets [6,66], fuzzy sets [5,42], interval analysis [67,68], neighbourhood systems [45,48], granular neutrosophic sets [69,70], etc. can be interpreted as different realizations of granulation mechanisms [8,71]. The core components of GrC include:

— formation of information granules,
— organization of granules into hierarchical levels, and
— granule-based approximation and reasoning.

Yao *et al.* [46] emphasized that granulation is fundamentally linked to the notion of neighbourhoods, where granules arise from local contexts surrounding objects. This observation establishes a natural conceptual bridge between GrC and topological or proximity-based structures. In particular, neighbourhood systems induce coverings of the universe that can be interpreted as granular structures at different resolutions.

Pedrycz & Bargiela [8] further highlighted the importance of multi-granularity, where information is analysed simultaneously at coarse and fine levels, enabling flexible modelling of complex systems such as social networks [72], decision processes [44] and cognitive systems [73]. These ideas strongly suggest that soft topological spaces, equipped with parameter-dependent neighbourhood mappings, provide an appropriate and mathematically robust environment for GrC. This perspective motivates the development of soft topological granular structures, where τ-neighbourhoods act as primitive granules, reachability induces higher-level granules, interior, closure and boundary characterize granular certainty and uncertainty.

**Figure 1.**
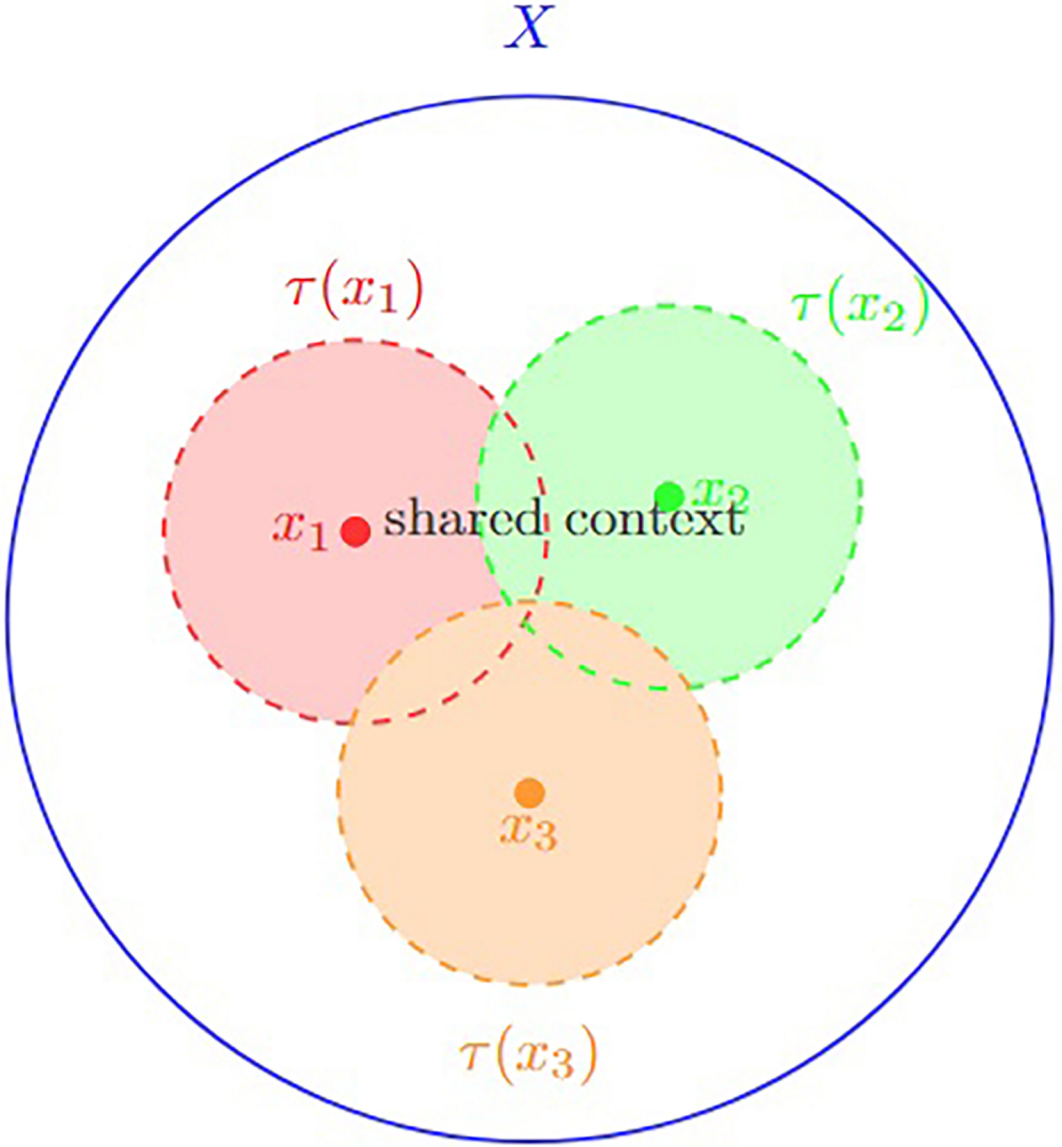
Soft topological granulation of the universe X in a specific granule via τ-neighbourhoods. Each coloured τ(x) represents a local information granule, while overlapping regions capture shared contextual or multi-criteria interactions among elements.

Figure 1 illustrates the conceptual relationship between soft topology and GrC adopted in this work. It depicts the soft topological granulation of the universe X induced by the collection of τ-neighbourhoods. Each coloured region, namely $\tau (x_{1})$, $\tau (x_{2})$ and $\tau (x_{3})$, represents a local information granule centred at an element of X. These granules are not necessarily disjoint; instead, their intersections indicate that certain elements simultaneously belong to multiple neighbourhoods and hence satisfy several contextual or attribute-based criteria. Consequently, the overlapping regions capture shared information and reveal potential interactions among different local contexts. From the viewpoint of GrC, the figure illustrates how a soft topology decomposes the universe into a family of interconnected information granules, where each neighbourhood provides a localized representation of the underlying structure, while the overlaps encode relational dependencies and multi-criteria associations among the elements of X.

## 3. Soft topological framework for granular computing

In this section, we develop a GrC framework grounded in the theory of soft topological spaces. Unlike classical granular models, the proposed framework employs proximity mappings as primitive neighbourhood operators. This approach allows information granules to be generated in a parameter independent yet context-sensitive manner, consistent with Molodtsov's original soft topology formulation. The resulting framework supports multi-level granulation, granular approximation and connectivity analysis within a unified mathematical setting.

### 3.1. Soft topological granular structure

Let (X,τ) be a soft topological space endowed with a proximity mapping $\tau :X\to 2^{X}$, where x∈τ(x) for all x∈X. Throughout the paper, all operators indexed by τ (such as $E_{\tau }$, $\rho_{\tau }$ and ${\overset{―}{A}}_{g}^{\tau })$ are understood to be generated by this fixed soft topological proximity mapping, and the meaning of these operators is summarized in table 2. We now construct a GrC structure on X using soft topological notions.

**Table 2.** Frequently used notation.

symbol	meaning
τ(x)	soft topological neighbourhood of x
$E_{\tau }(A)$	granular aggregation operator of A
$E_{\tau }^{k}(A)$	kth level granular aggregation operator of A
${\overset{―}{A}}_{g}^{\tau }$	granular closure of A
$\rho_{\tau }(x,y)$	granular distance or reachability depth between x and y
$I_{\tau }^{k}(A)$	kth step information or influence propagation operator
${∼}_{\tau }$	soft granular community
${\text{Core}}_{\tau }(A)$	granular core of A
${\text{Bnd}}_{\tau }(A)$	granular boundary of A
${\text{Per}}_{\tau }(A)$	granular periphery of A
GS(X,τ)	soft topological granular structure

**Definition 3.1.**

For each x∈X, the set τ(x) is called the primitive information granule generated by x. The family

$$G_{0}(X,\tau )=\{\tau (x):x\in X\}$$

is called the level-0 granular system.

Let us consider the following example:

**Example 3.1.**

Let $X=\{x_{1},x_{2},x_{3},x_{4},x_{5},x_{6},x_{7},x_{8},x_{9},x_{10}\}$ and define the proximity mapping $\tau :X\to 2^{X}$ by $\tau (x_{1})=\{x_{1},x_{2},x_{3}\},$$\tau (x_{2})=\{x_{1},x_{2},x_{4}\},$$\tau (x_{3})=\{x_{1},x_{3},x_{5}\},\tau (x_{4})=\{x_{2},$$x_{4},x_{6}\},\tau (x_{5})=\{x_{3},x_{5},x_{7}\},$$\tau (x_{6})=$$\{x_{4},x_{6},x_{8}\},\tau (x_{7})=\{x_{5},x_{7},x_{9}\},$$\tau (x_{8})=\{x_{6},x_{8},x_{10}\},$$\tau (x_{9})=\{x_{7},x_{9}\}\;\text{and }\tau (x_{10})=\{x_{8},x_{10}\}$.

The primitive information granule generated by $x_{1}$ is $\tau (x_{1})=\{x_{1},x_{2},x_{3}\}$, whereas the primitive information granule generated by $x_{6}$ is $\tau (x_{6})=\{x_{4},x_{6},x_{8}\}$ and so on.

Hence, the level-0 granular system is $G_{0}(X,\tau )=\{\tau (x_{i}):1\leq i\leq 10\}$, that is,

$$\begin{array}{rl} G_{0}(X,\tau )=\{ & \{x_{1},x_{2},x_{3}\},\{x_{1},x_{2},x_{4}\},\{x_{1},x_{3},x_{5}\},\{x_{2},x_{4},x_{6}\},\\ & \{x_{3},x_{5},x_{7}\},\{x_{4},x_{6},x_{8}\},\{x_{5},x_{7},x_{9}\},\{x_{6},x_{8},x_{10}\},\{x_{7},x_{9}\},\{x_{8},x_{10}\}\}. \end{array}$$

Thus, $G_{0}(X,\tau )$ consists of all primitive information granules generated by the elements of X.

Primitive granules represent the finest local perception units in the system and serve as the atomic building blocks for higher-level granules. The soft neighbourhood τ(x) may be viewed as the immediate sphere of interaction surrounding the element x. In social network settings, it can represent the collection of agents directly connected to or influenced by x under the given criteria. More generally, it captures the local context in which x participates within the underlying soft topological structure.

**Definition 3.2.**

Define the operator $E_{\tau }:2^{X}⟶2^{X}\;\text{by}\;E_{\tau }(A)=\underset{x\in A}{⋃}\tau (x)$.

The operator $E_{\tau }$ aggregates local neighbourhoods and acts as a granular synthesis mechanism. It enlarges a subset A by incorporating the soft neighbourhoods of all its elements. Thus, $E_{\tau }(A)$ represents the immediate granular environment generated by A. One may regard it as the first layer of influence, interaction or information surrounding the set A.

**Example 3.2.**

Consider the soft topological space (X,τ) defined in example 3.1 and let $A=\{x_{1},x_{4},x_{7}\}$. Then, $E_{\tau }(A)=\tau (x_{1})\cup \tau (x_{4})\cup \tau (x_{7})$.

Since $\tau (x_{1})=\{x_{1},x_{2},x_{3}\},\tau (x_{4})=\{x_{2},x_{4},x_{6}\}$ and $\tau (x_{7})=\{x_{5},x_{7},x_{9}\}$, we obtain

$$\begin{array}{rl} E_{\tau }(A) & =\{x_{1},x_{2},x_{3}\}\cup \{x_{2},x_{4},x_{6}\}\cup \{x_{5},x_{7},x_{9}\}\\ & =\{x_{1},x_{2},x_{3},x_{4},x_{5},x_{6},x_{7},x_{9}\}. \end{array}$$

Therefore, $E_{\tau }(\{x_{1},x_{4},x_{7}\})=\{x_{1},x_{2},x_{3},x_{4},x_{5},x_{6},x_{7},x_{9}\}$. This shows that the operator $E_{\tau }$ enlarges the set $\left\{x_{1},x_{4},x_{7}\right\}$ by incorporating the primitive information granules of all its elements.

**Remark 3.1.**

For any A⊆X, $A\subseteq E_{\tau }(A)$.

*Proof*. Let x∈A. Since (X,τ) is a soft topological space endowed with a proximity mapping τ, we have x∈τ(x). Therefore, x belongs to $\underset{y\in A}{⋃}\tau (y)=E_{\tau }(A).$ Hence, every element of *A* belongs to $E_{\tau }(A)$, and thus $A\subseteq E_{\tau }(A).$

**Definition 3.3.**

For any A⊆X, define inductively

$$E_{\tau }^{0}(A)=A,\;E_{\tau }^{k+1}(A)=E_{\tau }(E_{\tau }^{k}(A)),\;k\geq 0.$$

The family

$$G_{k}(A)=\{E_{\tau }^{k}(\{x\}):x\in A\}$$

is called the kth level granular system induced by A. The iterated operator $E_{\tau }^{k}(A)$ describes the progressive expansion of a granule through repeated neighbourhood aggregation. Each iteration allows influence or information to spread one step further. Consequently, $E_{\tau }^{k}(A)$ represents the region that can be reached from A after k successive stages of interaction. Consider the following example to illustrate the definition.

**Example 3.3.**

Consider the soft topological space (X,τ) defined in example 3.1 and let $A=\{x_{1}\}$. By definition, $E_{\tau }^{0}(A)=A=\{x_{1}\}$. Since $\tau (x_{1})=\{x_{1},x_{2},x_{3}\}$, we obtain $E_{\tau }^{1}(A)=E_{\tau }(\{x_{1}\})=\{x_{1},x_{2},x_{3}\}$.

Next,

$$\begin{array}{rl} E_{\tau }^{2}(A) & =E_{\tau }(E_{\tau }^{1}(A))\\ & =E_{\tau }(\{x_{1},x_{2},x_{3}\})\\ & =\tau (x_{1})\cup \tau (x_{2})\cup \tau (x_{3})\\ & =\{x_{1},x_{2},x_{3}\}\cup \{x_{1},x_{2},x_{4}\}\cup \{x_{1},x_{3},x_{5}\}\\ & =\{x_{1},x_{2},x_{3},x_{4},x_{5}\}. \end{array}$$

Similarly,

$$\begin{array}{rl} E_{\tau }^{3}(A) & =E_{\tau }(\{x_{1},x_{2},x_{3},x_{4},x_{5}\})\\ & =\tau (x_{1})\cup \tau (x_{2})\cup \tau (x_{3})\cup \tau (x_{4})\cup \tau (x_{5})\\ & =\{x_{1},x_{2},x_{3},x_{4},x_{5},x_{6},x_{7}\}. \end{array}$$

Hence, the granule generated by $\left\{x_{1}\right\}$ expands progressively as $\left\{x_{1}\}\subseteq \{x_{1},x_{2},x_{3}\}\subseteq \{x_{1},x_{2},x_{3},x_{4},x_{5}\}\subseteq \{x_{1},x_{2},x_{3},x_{4},x_{5},x_{6},x_{7}\right\}$.

Moreover, the second level granular system induced by $A=\{x_{1}\}$ is $G_{2}(A)=\{E_{\tau }^{2}(\{x_{1}\})\}=\{\{x_{1},x_{2},x_{3},x_{4},x_{5}\}\}$.

**Definition 3.4.**

Let (X,τ) be a soft topological space endowed with a proximity mapping $\tau :X\to 2^{X}$. A subset C⊆X is called τ-closed if $E_{\tau }(C)\subseteq C.$

**Example 3.4.**

Consider the soft topological space (X,τ) defined in example 3.1 and let $C=\{x_{7},x_{8},x_{9},x_{10}\}$.

Then,

$$\begin{array}{rl} E_{\tau }(C) & =\tau (x_{7})\cup \tau (x_{8})\cup \tau (x_{9})\cup \tau (x_{10})\\ & =\{x_{5},x_{7},x_{9}\}\cup \{x_{6},x_{8},x_{10}\}\cup \{x_{7},x_{9}\}\cup \{x_{8},x_{10}\}\\ & =\{x_{5},x_{6},x_{7},x_{8},x_{9},x_{10}\}. \end{array}$$

Since $E_{\tau }(C)=\{x_{5},x_{6},x_{7},x_{8},x_{9},x_{10}\}⊈C$, the set C is not τ-closed. On the other hand, consider D=X. Then, $E_{\tau }(D)=\underset{x\in X}{⋃}\tau (x)=X$. Therefore, $E_{\tau }(D)\subseteq D$, and hence D is a τ-closed subset of X.

**Definition 3.5.**

A subset A⊆X is called granularly saturated if $E_{\tau }(A)=A.$ If a smallest granularly saturated set containing A exists, it is called the granular closure of A and is denoted by ${\overset{―}{A}}_{g}^{\;\tau }.$

Granular saturation may capture the notion of stable information regions. Once such a set is reached, further neighbourhood aggregation does not generate any new elements. It may therefore be viewed as a self-contained granule that has fully absorbed its surrounding influence. The granular closure of a set *A* is the smallest stable granular region containing *A*. It describes the ultimate extent of the influence generated by *A* after all possible neighbourhood expansions have taken place. The following example illustrates the existence of granularly saturated sets in a soft topological space.

**Example 3.5.**

Let $X=\{x_{1},x_{2},x_{3},x_{4},x_{5},x_{6},x_{7},x_{8},x_{9},x_{10}\}$ and define the proximity mapping $\tau :X\to 2^{X}$ by $\tau (x_{1})=\{x_{1},x_{2},x_{3}\},$$\tau (x_{2})=\{x_{1},x_{2},x_{4}\},$$\tau (x_{3})=\{x_{1},x_{3},x_{5}\},$$\tau (x_{4})=\{x_{2},x_{4},x_{6}\},$$\tau (x_{5})=\{x_{3},x_{5},x_{7}\},$$\tau (x_{6})=$$\{x_{4},x_{6},x_{8}\},$$\tau (x_{7})=\{x_{7},x_{8},x_{9}\},$$\tau (x_{8})=\{x_{7},x_{8},x_{10}\},$$\tau (x_{9})=\{x_{7},x_{9}\}\;\text{and}\;\tau (x_{10})=\{x_{8},x_{10}\}$.

Let $A=\{x_{9}\}$. Then, $E_{\tau }(A)=E_{\tau }(\{x_{9}\})=\tau (x_{9})=\{x_{7},x_{9}\}$.

Next,

$$\begin{array}{rl} E_{\tau }^{2}(A) & =E_{\tau }(\{x_{7},x_{9}\})\\ & =\tau (x_{7})\cup \tau (x_{9})\\ & =\{x_{7},x_{8},x_{9}\}\cup \{x_{7},x_{9}\}\\ & =\{x_{7},x_{8},x_{9}\}. \end{array}$$

Similarly,

$$\begin{array}{rl} E_{\tau }^{3}(A) & =E_{\tau }(\{x_{7},x_{8},x_{9}\})\\ & =\tau (x_{7})\cup \tau (x_{8})\cup \tau (x_{9})\\ & =\{x_{7},x_{8},x_{9}\}\cup \{x_{7},x_{8},x_{10}\}\cup \{x_{7},x_{9}\}\\ & =\{x_{7},x_{8},x_{9},x_{10}\}. \end{array}$$

Now,

$$\begin{array}{rl} E_{\tau }(\{x_{7},x_{8},x_{9},x_{10}\}) & =\tau (x_{7})\cup \tau (x_{8})\cup \tau (x_{9})\cup \tau (x_{10})\\ & =\{x_{7},x_{8},x_{9}\}\cup \{x_{7},x_{8},x_{10}\}\cup \{x_{7},x_{9}\}\cup \{x_{8},x_{10}\}\\ & =\{x_{7},x_{8},x_{9},x_{10}\}. \end{array}$$

Therefore, $E_{\tau }(\{x_{7},x_{8},x_{9},x_{10}\})=\{x_{7},x_{8},x_{9},x_{10}\}$, and hence the set $\left\{x_{7},x_{8},x_{9},x_{10}\right\}$ is granularly saturated. Since $\left\{x_{9}\}\subseteq \{x_{7},x_{8},x_{9},x_{10}\right\}$ and no proper granularly saturated subset containing $x_{9}$ exists, we conclude that ${\overset{―}{\left\{x_{9}\right\}}}_{g}^{\;\tau }=\{x_{7},x_{8},x_{9},x_{10}\}$.

Thus, the granular closure of $\left\{x_{9}\right\}$ is the smallest stable granular region generated by repeated neighbourhood aggregation starting from $x_{9}$.

**Remark 3.2.**

Let (X,τ) be a soft topological space endowed with a proximity mapping $\tau :X\to 2^{X}$. Then, any granularly saturated set A⊆X is τ-closed and vice versa.

*Proof*. Suppose A is granularly saturated. Then, $E_{\tau }(A)=A.$ Hence, $E_{\tau }(A)\subseteq A$. Thus, by definition 3.4, *A* is τ-closed.

Conversely, suppose *A* is τ-closed; then, $E_{\tau }(A)\subseteq A$. By remark 3.1, $A\subseteq E_{\tau }(A)$. Therefore, $A=E_{\tau }(A)$, which shows that *A* is granularly saturated.

**Proposition 3.1.**

The granular closure ${\overset{―}{A}}_{g}^{\;\tau }$ is τ-closed.

*Proof*. Let C be the family of all τ-closed sets containing A. Then,

$${\overset{―}{A}}_{g}^{\;\tau }=\underset{C\in C}{⋂}C.$$

For any $x\in {\overset{―}{A}}_{g}^{\;\tau }$, we have x∈C for all C∈C. Since each C is τ-closed, τ(x)⊆C for all C∈C. Hence,

$$\tau (x)\subseteq \underset{C\in C}{⋂}C={\overset{―}{A}}_{g}^{\;\tau }.$$

Therefore,

$$E_{\tau }({\overset{―}{A}}_{g}^{\;\tau })\subseteq {\overset{―}{A}}_{g}^{\;\tau },$$

and ${\overset{―}{A}}_{g}^{\;\tau }$ is τ-closed.

**Definition 3.6.**

Define the function $\rho_{\tau }:X\times X\to N\cup \{\infty \}$ by $\rho_{\tau }(x,y)=\inf\{k\geq 0:y\in E_{\tau }^{k}(\{x\})\},$ with the convention that inf∅=∞.

The value $\rho_{\tau }(x,y)$ represents the granular distance or reachability depth between x and y. It measures how far *y* is from *x* in terms of granular expansion. Rather than measuring geometric distance, it counts the minimum number of neighbourhood-propagation steps required for information originating at *x* to reach *y*.

**Example 3.6.**

Consider the soft topological space (X,τ) defined in example 3.5 and consider the element $x_{1}$. Since $E_{\tau }^{0}(\{x_{1}\})=\{x_{1}\}$, we have $\rho_{\tau }(x_{1},x_{1})=0$. Moreover, $E_{\tau }^{1}(\{x_{1}\})=\{x_{1},x_{2},x_{3}\}$, and therefore $\rho_{\tau }(x_{1},x_{2})=1\;\text{and}\;\rho_{\tau }(x_{1},x_{3})=1$.

Next, $E_{\tau }^{2}(\{x_{1}\})=\{x_{1},x_{2},x_{3},x_{4},x_{5}\}$, which gives $\rho_{\tau }(x_{1},x_{4})=2\;\text{and}\;\rho_{\tau }(x_{1},x_{5})=2$. Similarly, $E_{\tau }^{3}(\{x_{1}\})=\{x_{1},x_{2},x_{3},x_{4},x_{5},x_{6},x_{7}\}$, and hence $\rho_{\tau }(x_{1},x_{6})=3\;\text{and}\;\rho_{\tau }(x_{1},x_{7})=3$. Furthermore, $E_{\tau }^{4}(\{x_{1}\})=\{x_{1},x_{2},x_{3},x_{4},x_{5},x_{6},x_{7},x_{8},x_{9}\}$, so that $\rho_{\tau }(x_{1},x_{8})=4\;\text{and}\;\rho_{\tau }(x_{1},x_{9})=4$. Finally, $E_{\tau }^{5}(\{x_{1}\})=X$, which implies that $\rho_{\tau }(x_{1},x_{10})=5$. Thus, the granular distance measures the minimum number of neighbourhood aggregation steps required for information originating at $x_{1}$ to reach another element of X.

**Definition 3.7.**

For A⊆X and k∈N, define

$$I_{\tau }^{k}(A)=\{y\in X:\exists \;x\in A\text{ such that }\rho_{\tau }(x,y)\leq k\}.$$

This operator models k-step information or influence propagation. It models the spread of information, influence or activity originating from *A* after *k* stages of propagation. It captures the dynamic evolution of interaction processes within the granular structure. Consider the following illustrative example:

**Example 3.7.**

Let (X,τ) be the soft topological space defined in example 3.5 and let $A=\{x_{1}\}$. For k=2, we have $I_{\tau }^{2}(A)=\{y\in X:\rho_{\tau }(x_{1},y)\leq 2\}$. From example 3.5, $\rho_{\tau }(x_{1},x_{1})=0,\;\rho_{\tau }(x_{1},x_{2})=1,\;\rho_{\tau }(x_{1},x_{3})=1$, $\rho_{\tau }(x_{1},x_{4})=2\;\text{and}\;\rho_{\tau }(x_{1},x_{5})=2$. Therefore, $I_{\tau }^{2}(\{x_{1}\})=\{x_{1},x_{2},x_{3},x_{4},x_{5}\}$. Similarly, $I_{\tau }^{4}(\{x_{1}\})=\{y\in X:\rho_{\tau }(x_{1},y)\leq 4\}=\{x_{1},x_{2},x_{3},x_{4},x_{5},x_{6},x_{7},x_{8},x_{9}\}$.

Thus, after two stages of propagation, information originating from $x_{1}$ reaches precisely the elements $\left\{x_{1},x_{2},x_{3},x_{4},x_{5}\right\}$, whereas after four stages it reaches all elements of X except $x_{10}$.

**Definition 3.8.**

Define a relation ${∼}_{\tau }$ on X by

$$x{∼}_{\tau }y\;⟺\;\rho_{\tau }(x,y)<\infty \;\text{and}\;\rho_{\tau }(y,x)<\infty .$$

The equivalence classes of ${∼}_{\tau }$ are called soft granular communities. The relation ${∼}_{\tau }$ identifies elements that belong to the same coherent granular community. Members of such a community exhibit strong mutual reachability and share common patterns of interaction within the soft topological environment.

**Example 3.8.**

Let (X,τ) be the soft topological space defined in example 3.5 and consider the subset $C=\{x_{7},x_{8},x_{9},x_{10}\}$.

From example 3.5, we know that $E_{\tau }(C)=C$. Hence, every element of C can reach every other element of C in finitely many neighbourhood aggregation steps.

For example, $\rho_{\tau }(x_{9},x_{7})=1,\;\rho_{\tau }(x_{9},x_{8})=2,\;\rho_{\tau }(x_{9},x_{10})=3$, $\rho_{\tau }(x_{10},x_{8})=1,\;\rho_{\tau }(x_{10},x_{7})=2\;\text{and}\;\rho_{\tau }(x_{10},x_{9})=3$. Therefore, $\rho_{\tau }(x,y)<\infty \;\text{and}\;\rho_{\tau }(y,x)<\infty$ for all x,y∈C. Consequently, $x{∼}_{\tau }y\;\text{for all }x,y\in C$. Hence, $C=\{x_{7},x_{8},x_{9},x_{10}\}$ is a soft granular community.

**Definition 3.9.**

Let C be any subset of X. Then,

— The granular core of C is defined as ${\text{Core}}_{\tau }(C)=\{x\in C:\tau (x)\subseteq C\}.$
— The granular boundary of C is defined as ${\text{Bnd}}_{\tau }(C)=\{x\in C:\tau (x)\cap (X\setminus C)\neq \emptyset \}.$
— The granular periphery of C is defined as ${\text{Per}}_{\tau }(C)=E_{\tau }(C)\setminus C.$

If we consider C to be a soft granular community, the granular core may consists of the most central and structurally stable members of a community. These elements play a dominant role in maintaining the integrity of the community and typically remain influential under repeated propagation processes. The granular boundary forms the transitional zone between the core of a community and its external surroundings. Elements in the boundary often act as intermediaries through which information, influence or interactions pass between different communities. The granular periphery contains the outermost members of a community. These elements are weakly connected to the central structure and generally exert limited influence on the overall behaviour of the granular community.

**Example 3.9.**

Consider the soft granular community $C=\{x_{7},x_{8},x_{9},x_{10}\}$ of example 3.5. For every x∈C, we have τ(x)⊆C. Indeed, $\tau (x_{7})=\{x_{7},x_{8},x_{9}\}\subseteq C$, $\tau (x_{8})=\{x_{7},x_{8},x_{10}\}\subseteq C$, $\tau (x_{9})=\{x_{7},x_{9}\}\subseteq C$ and $\tau (x_{10})=\{x_{8},x_{10}\}\subseteq C$.

Therefore, ${\text{Core}}_{\tau }(C)=\{x_{7},x_{8},x_{9},x_{10}\}=C.$ Since no element of C has a neighbourhood intersecting $X\setminus C=\{x_{1},x_{2},x_{3},x_{4},x_{5},x_{6}\}$, we obtain ${\text{Bnd}}_{\tau }(C)=\emptyset$. Moreover, ${\text{Per}}_{\tau }(C)=E_{\tau }(C)\setminus C=C\setminus C=\emptyset$.

Thus, the community $C=\{x_{7},x_{8},x_{9},x_{10}\}$ is completely self-contained, i.e. every member belongs to the granular core, and the community possesses neither a granular boundary nor a granular periphery.

**Definition 3.10.**

Let (X,τ) be a soft topological space. A family G of non-empty subsets of X is called a granular system induced by τ if

G={G⊆X:G=τ(x) for some x∈X}orG⊆P(X)

satisfies the following conditions:

(G1) $\underset{G\in G}{⋃}G=X,$
(G2) For every G∈G and every x∈G, there exists H∈G such that x∈H⊆G,
(G3) For all x,y∈X, if y∈τ(x), then τ(y)∩τ(x)≠∅.

Each element G∈G is called a soft granule.

**Definition 3.11.**

For two granular systems $G_{i}$ and $G_{j}$, define

$$G_{i}⪯G_{j}\;⟺\;\forall G\in G_{i},\exists H\in G_{j}\text{ such that }G\subseteq H.$$

Then, the tuple $\mathrm{GS}(X,\tau )=(X,\;G,\;E_{\tau },\;\rho_{\tau },\;I_{\tau },\;{∼}_{\tau },\;⪯)$ is called a soft topological granular structure with the granular system G.

The soft topological granular structure provides the complete environment in which granular interactions take place. It combines local neighbourhood information, aggregation mechanisms, reachability relations and community structures into a unified framework for studying hierarchical organization and information flow.

**Example 3.10.**

Let $X=\{x_{1},x_{2},x_{3},x_{4}\}$ and let (X,τ) be a soft topological space defined by the soft neighbourhood mapping $\tau (x_{1})=\{x_{1},x_{2}\},\;\tau (x_{2})=\{x_{2}\},\;\tau (x_{3})=\{x_{3},x_{4}\}\;\text{and }\tau (x_{4})=\{x_{3},x_{4}\}$. The induced granular system $G_{0}=\{\tau (x_{1}),\tau (x_{2}),\tau (x_{3}),\tau (x_{4})\}=\{\{x_{1},x_{2}\},\{x_{2}\},\{x_{3},x_{4}\},\{x_{3},x_{4}\}\}$ forms a family of overlapping granules that covers X. Each granule represents local contextual information centred at a node, whereas overlaps capture shared attributes or interactions. Moreover, $G_{0}$ satisfies

(G1) $\underset{G\in G_{0}}{⋃}G=\tau (x_{1})\cup \tau (x_{2})\cup \tau (x_{3})\cup \tau (x_{4})=\{x_{1},x_{2}\}\cup \{x_{2}\}\cup \{x_{3},x_{4}\}\cup \{x_{3},x_{4}\}=X$.
(G2) For $G\in G_{0}$,
  if $x_{1}\in G=\tau (x_{1})=\{x_{1},x_{2}\}$, we have $\tau (x_{1})=\{x_{1},x_{2}\}\subseteq \tau (x_{1})=\{x_{1},x_{2}\}$,
  if $x_{2}\in G=\tau (x_{1})=\{x_{1},x_{2}\}$, we have $\tau (x_{2})=\{x_{2}\}\subseteq \tau (x_{1})=\{x_{1},x_{2}\}$,
  if $x_{2}\in G=\tau (x_{2})=\{x_{2}\}$, we have $\tau (x_{2})=\{x_{2}\}\subseteq \tau (x_{2})=\{x_{2}\}$,
  if $x_{3}\in G=\tau (x_{3})=\{x_{3},x_{4}\}$, we have $\tau (x_{3})=\{x_{3},x_{4}\}\subseteq \tau (x_{3})=\{x_{3},x_{4}\}$,
  if $x_{4}\in G=\tau (x_{3})=\{x_{3},x_{4}\}$, we have $\tau (x_{4})=\{x_{3},x_{4}\}\subseteq \tau (x_{3})=\{x_{3},x_{4}\}$,
  if $x_{3}\in G=\tau (x_{4})=\{x_{3},x_{4}\}$, we have $\tau (x_{3})=\{x_{3},x_{4}\}\subseteq \tau (x_{4})=\{x_{3},x_{4}\}$ and
  if $x_{4}\in G=\tau (x_{4})=\{x_{3},x_{4}\}$, we have $\tau (x_{4})=\{x_{3},x_{4}\}\subseteq \tau (x_{4})=\{x_{3},x_{4}\}$.
(G3) If y∈τ(x), then τ(x)∩τ(y)≠∅, since y∈τ(y) for all y.

Hence, $G_{0}$ is a granular system induced by the soft topology τ.

**Figure 2.**
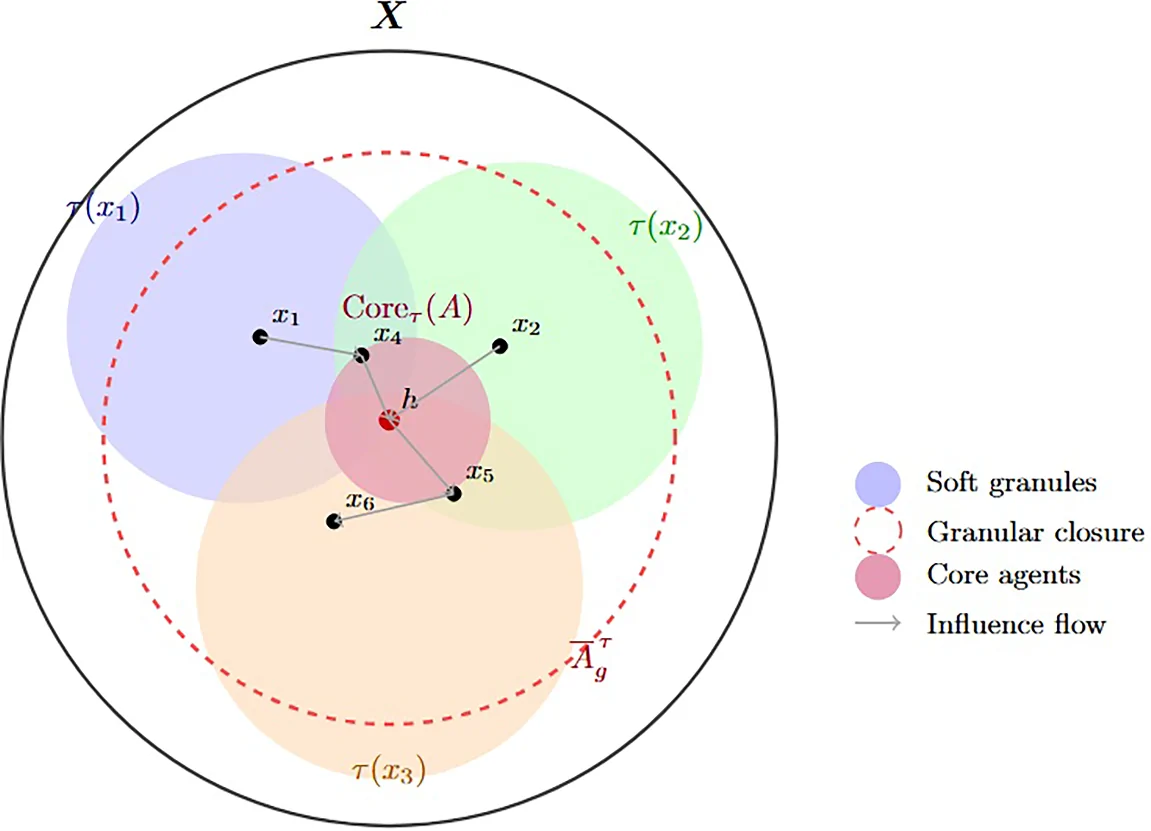
Soft topological granular structure of a multi-criteria social network. Overlapping coloured regions represent soft granules induced by τ-neighbourhoods. Influence propagates through local neighbourhoods and stabilizes into the granular closure ${\overset{―}{A}}_{g}^{\;\tau }$, with a dense core of structurally influential agents.

To provide a unified visual intuition for the proposed soft topological granular structure and its associated operators, we present a conceptual illustration in figure 2 that depicts granulation, influence propagation, stabilization and community formation within a single framework, which will be discussed in the later sections. It illustrates the soft topological granular structure of a multi-criteria social network induced by the family of τ-neighbourhoods. The overlapping coloured regions represent soft information granules, where each granule consists of agents that are locally related through one or more social attributes, preferences or interaction criteria. The intersections among these granules indicate that certain agents participate simultaneously in multiple social contexts, and thereby act as bridges connecting different communities. Influence propagates through these local neighbourhoods by successive granular interactions, gradually extending beyond the initially activated set of agents. This diffusion process eventually stabilizes in the granular closure ${\overset{―}{A}}_{g}^{\;\tau }$, which represents the collection of all agents that become reachable under repeated neighbourhood expansion. The dense central region corresponds to a structurally influential core of agents whose extensive interconnections enable them to play a significant role in maintaining cohesion and facilitating the spread of information throughout the network.

The granular structure introduced above is motivated by the need for a mathematically coherent and flexible foundation for GrC that is capable of handling context dependence, multi-level abstraction and uncertainty without relying on metric or probabilistic assumptions. By interpreting soft topological neighbourhoods as primitive information granules, the proposed framework naturally aligns with the neighbourhood-based philosophy of GrC emphasized in the literature. The expansion operator $E_{\tau }$ and its iterates formalize the process of granule aggregation and hierarchical granulation, whereas granular saturation captures stable information regions that persist under contextual expansion. The reachability function and influence operator $I_{\tau }$ provide a quantitative and structural mechanism for modelling information propagation across granules, which is essential for analysing interaction and influencing dynamics in complex systems. Furthermore, the induced granular equivalence relation and associated core-boundary periphery decomposition enable the identification of coherent information communities and their internal stability and external exposure. Collectively, these notions establish a unified soft topological environment in which granule formation, refinement, approximation and connectivity can be rigorously studied, and thus it extends GrC into a topologically grounded and analytically tractable framework.

### 3.2. Properties of soft topological granular structures

Throughout this section, we assume that (X,τ) is a soft topological space, and the proximity mappings are assumed to be such that x∈τ(x),∀x∈X, and GS(X,τ) represent the associated soft topological granular structure.

**Proposition 3.2.**

For all subsets A,B⊆X, if A⊆B, then $E_{\tau }(A)\subseteq E_{\tau }(B).$

*Proof*. Assume A⊆B. Let $y\in E_{\tau }(A)$. Then, there exists x∈A such that y∈τ(x). Since A⊆B, we have x∈B. Hence, $y\in \underset{x\in B}{⋃}\tau (x)=E_{\tau }(B)$. Thus, $E_{\tau }(A)\subseteq E_{\tau }(B)$.

**Theorem 3.1.**

For any A⊆X, the sequence $(A,E_{\tau }(A),E_{\tau }^{2}(A),\cdots E_{\tau }^{k}(A))$ is monotonically increasing with respect to set inclusion, i.e. $A\subseteq E_{\tau }(A)\subseteq E_{\tau }^{2}(A)\subseteq \cdots E_{\tau }^{k}(A)$.

*Proof*. We proceed by induction on k.

For k=0, $A\subseteq E_{\tau }(A)$ since x∈τ(x) for all x∈X. Assume $E_{\tau }^{k}(A)\subseteq E_{\tau }^{k+1}(A)$. Applying $E_{\tau }$ to both sides and using proposition 3.2,

$$E_{\tau }^{k+1}(A)=E_{\tau }(E_{\tau }^{k}(A))\subseteq E_{\tau }(E_{\tau }^{k+1}(A))=E_{\tau }^{k+2}(A).$$

Thus, the sequence is increasing.

**Theorem 3.2.**

For every subset A⊆X, the granular closure ${\overset{―}{A}}_{g}^{\;\tau }$ exists and is unique.

*Proof*. Consider the increasing sequence $\{E_{\tau }^{k}(A){\}}_{k\geq 0}$. Define

$$C=\overset{\infty }{\underset{k=0}{⋃}}E_{\tau }^{k}(A).$$

We show that C is granularly saturated.

By monotonicity, $E_{\tau }(C)=E_{\tau }\;\left(\underset{k}{⋃}E_{\tau }^{k}(A)\right)=\underset{k}{⋃}E_{\tau }^{k+1}(A)=C.$ Thus C is granularly saturated. To establish minimality, let S⊆X be any granularly saturated subset such that A⊆S. Since S is granularly saturated, $E_{\tau }(S)=S$. By monotonicity of the operator $E_{\tau }$,

$$E_{\tau }(A)\subseteq E_{\tau }(S)=S.$$

Applying $E_{\tau }$ repeatedly and using induction, we obtain

$$E_{\tau }^{k}(A)\subseteq S\;\text{for all }k\in N.$$

Taking the union over all k yields

$${\overset{―}{A}}_{g}^{\;\tau }=\overset{\infty }{\underset{k=0}{⋃}}E_{\tau }^{k}(A)\subseteq S.$$

Hence, ${\overset{―}{A}}_{g}^{\;\tau }$ is contained in every granularly saturated set containing A. Hence, $C={\overset{―}{A}}_{g}^{\;\tau }$.

**Theorem 3.3.**

Let (X,τ) be a soft topological space and let A⊆X. Let $\{A_{k}{\}}_{k\in N}$ be the sequence defined by $A_{0}=A,A_{k+1}=E_{\tau }(A_{k}).$ Then, there exists $k_{0}\in N$ such that for all $k\geq k_{0}$, $A_{k}={\overset{―}{A}}_{g}^{\;\tau },$ where ${\overset{―}{A}}_{g}^{\;\tau }$ denotes the granular closure of A.

*Proof*.
$\{A_{k}{\}}_{k\in N}$ is a monotone increasing sequence by theorem 3.1, with respect to set inclusion.

Next, we show by induction that $A_{k}\subseteq {\overset{―}{A}}_{g}^{\;\tau }\;\text{for all }k.$ For k=0, this holds since $A\subseteq {\overset{―}{A}}_{g}^{\;\tau }$. Assume $A_{k}\subseteq {\overset{―}{A}}_{g}^{\;\tau }$. From proposition 3.1, since ${\overset{―}{A}}_{g}^{\;\tau }$ is τ-closed, hence $E_{\tau }({\overset{―}{A}}_{g}^{\;\tau })\subseteq {\overset{―}{A}}_{g}^{\;\tau }.$ By monotonicity of $E_{\tau }$, we have

$$E_{\tau }(A_{k})\subseteq E_{\tau }({\overset{―}{A}}_{g}^{\;\tau })\subseteq {\overset{―}{A}}_{g}^{\;\tau }.$$

Hence,

$$A_{k+1}\subseteq {\overset{―}{A}}_{g}^{\;\tau }.$$

Now, since $\left\{A_{k}\right\}$ is increasing and bounded above by ${\overset{―}{A}}_{g}^{\;\tau }$, there exists a least $k_{0}\in N$ such that

$$A_{k_{0}}=A_{k_{0}+1}=E_{\tau }(A_{k_{0}}).$$

Then,

$$E_{\tau }(A_{k_{0}})\subseteq A_{k_{0}},$$

so $A_{k_{0}}$ is τ-closed.

Because $A_{k_{0}}$ is τ-closed and contains A, and ${\overset{―}{A}}_{g}^{\;\tau }$ is the smallest such set, so we have

$${\overset{―}{A}}_{g}^{\;\tau }\subseteq A_{k_{0}}.$$

Again, since $A_{k}\subseteq {\overset{―}{A}}_{g}^{\;\tau }\;\text{for all }k,$ we must have $A_{k_{0}}={\overset{―}{A}}_{g}^{\;\tau }.$ Thus, by monotonicity, $A_{k}=A_{k_{0}}$ for all $k\geq k_{0}$.

**Theorem 3.4.**

Let (X,τ) be a soft topological space endowed with a proximity mapping τ. Let A⊆X be a granularly saturated set, i.e. $E_{\tau }(A)\subseteq A$. Assume $E_{\tau }^{0}(A)=A$ and let a,b∈A. Then, $b\in {\overset{―}{\left\{a\right\}}}_{g}^{\;\tau }\cap A⟺b\in {\text{Reach}}_{A}(X,\tau ,a).$

*Proof*. Assume that $b\in {\overset{―}{\left\{a\right\}}}_{g}^{\;\tau }\cap A.$ By definition of the granular closure operator,

$${\overset{―}{\left\{a\right\}}}_{g}^{\;\tau }=\overset{\infty }{\underset{n=0}{⋃}}E_{\tau }^{\;n}(\{a\}),$$

hence by the same analogy as in theorem 3.3, there exists an integer n≥0 such that $b\in E_{\tau }^{\;n}(\{a\}).$ Now, we consider two cases.

*Case 1: n=0.*

If n=0, then $E_{\tau }^{\;0}(\{a\})=\{a\}$, and therefore b=a. The trivial sequence (a) is a τ-path from a to itself lying entirely in A. Hence, $b\in {\text{Reach}}_{A}(X,\tau ,a).$

*Case 2: n≥1.*

By definition of the expansion operator,

$$E_{\tau }^{\;n}(\{a\})=E_{\tau }(E_{\tau }^{\;n-1}(\{a\}))=\underset{x\in E_{\tau }^{\;n-1}(\{a\})}{⋃}\tau (x).$$

Thus, there exists an element $x_{n-1}\in E_{\tau }^{\;n-1}(\{a\})$ such that $b\in \tau (x_{n-1}).$ Applying the same argument recursively to $x_{n-1}$, we obtain a finite sequence $a=x_{0},x_{1},x_{2},\ldots ,x_{n-1},x_{n}=b$ such that $x_{i+1}\in \tau (x_{i}),\;i=0,1,\ldots ,n-1.$

By construction, each $x_{i}$ belongs to $E_{\tau }^{\;i}(\{a\})$ and hence lies in A as A is granularly saturated. Therefore, $\left(x_{0},x_{1},\ldots ,x_{n}\right)$ is a τ-path from a to b contained in A. This implies ${\text{Path}}_{A}(X,\tau ,a,b)\neq \emptyset ,$ and consequently $b\in {\text{Reach}}_{A}(X,\tau ,a).$

Now, assume that $b\in {\text{Reach}}_{A}(X,\tau ,a).$ By definition of reachability, there exists a τ-path $\left(x_{0},x_{1},\ldots ,x_{n}\right)$ such that $x_{0}=a,x_{n}=b,x_{i+1}\in \tau (x_{i}),x_{i}\in A\text{ for all }i.$

We show by induction on i that $x_{i}\in E_{\tau }^{\;i}(\{a\}),i=0,1,\ldots ,n.$ For i=0, we have $x_{0}=a\in E_{\tau }^{\;0}(\{a\})$. Assume that $x_{i}\in E_{\tau }^{\;i}(\{a\})$. Since $x_{i+1}\in \tau (x_{i})$, it follows that

$$x_{i+1}\in \underset{y\in E_{\tau }^{\;i}(\{a\})}{⋃}\tau (y)=E_{\tau }^{\;i+1}(\{a\}).$$

Thus, the claim holds for all i.

In particular, $b=x_{n}\in E_{\tau }^{\;n}(\{a\})\subseteq \overset{\infty }{\underset{k=0}{⋃}}E_{\tau }^{\;k}(\{a\})={\overset{―}{\left\{a\right\}}}_{g}^{\;\tau }.$ Since b∈A, we conclude that $b\in {\overset{―}{\left\{a\right\}}}_{g}^{\;\tau }\cap A.$

Hence, $b\in {\overset{―}{\left\{a\right\}}}_{g}^{\;\tau }\cap A⟺b\in {\text{Reach}}_{A}(X,\tau ,a),$ which completes the proof.

**Lemma 3.1.**

Let (X,τ) be a soft topological space and let C be an equivalence class of the relation ${∼}_{\tau }$. Then, for any $a\in C,C={\overset{―}{\left\{a\right\}}}_{g}^{\;\tau }.$

*Proof*. Let a∈C be fixed. Let x∈C. Since C is the equivalence class of a under ${∼}_{\tau }$, we have $a{∼}_{\tau }x,$ which means $\rho_{\tau }(a,x)<\infty \;\text{and}\;\rho_{\tau }(x,a)<\infty .$ In particular, $\rho_{\tau }(a,x)<\infty$ implies that there exists a finite τ-path from a to x. Now, by the above theorem, it is equivalent to $x\in {\overset{―}{\left\{a\right\}}}_{g}^{\;\tau },$ and therefore $C\subseteq {\overset{―}{\left\{a\right\}}}_{g}^{\;\tau }.$

Now, to prove ${\overset{―}{\left\{a\right\}}}_{g}^{\;\tau }\subseteq C$, let $x\in {\overset{―}{\left\{a\right\}}}_{g}^{\;\tau }$. By definition of granular closure,

$${\overset{―}{\left\{a\right\}}}_{g}^{\;\tau }=\overset{\infty }{\underset{n=0}{⋃}}E_{\tau }^{\;n}(\{a\}).$$

Hence, by the same analogy as in theorem 3.3, there exists an integer n≥0 such that $x\in E_{\tau }^{\;n}(\{a\}).$ This implies the existence of a finite τ-path from a to x, and therefore $\rho_{\tau }(a,x)<\infty$, that is, x∈C. Thus, ${\overset{―}{\left\{a\right\}}}_{g}^{\;\tau }\subseteq C.$

Hence, we obtain $C={\overset{―}{\left\{a\right\}}}_{g}^{\;\tau },$ which completes the proof.

**Theorem 3.5.**

For x,y∈X, $\rho_{\tau }(x,y)<\infty$ if and only if $y\in {\overset{―}{\left\{x\right\}}}_{g}^{\;\tau }$.

*Proof*. If $\rho_{\tau }(x,y)=k$, then using theorem 3.2, $y\in E_{\tau }^{k}(\{x\})\subseteq {\overset{―}{\left\{x\right\}}}_{g}^{\;\tau }$. Conversely, if $y\in {\overset{―}{\left\{x\right\}}}_{g}^{\;\tau }$, then by construction in theorem 3.2, there exists k such that $y\in E_{\tau }^{k}(\{x\})$, hence $\rho_{\tau }(x,y)\leq k$.

**Lemma 3.2.**

Let (X,τ) be a soft topological space endowed with a proximity mapping τ. Let x,y∈X. If $y\in {\overset{―}{\left\{x\right\}}}_{g}^{\;\tau },$ then ${\overset{―}{\left\{y\right\}}}_{g}^{\;\tau }\subseteq {\overset{―}{\left\{x\right\}}}_{g}^{\;\tau }.$

*Proof*. Assume that $y\in {\overset{―}{\left\{x\right\}}}_{g}^{\;\tau }.$ By definition of the granular closure operator,

$${\overset{―}{\left\{x\right\}}}_{g}^{\;\tau }=\overset{\infty }{\underset{n=0}{⋃}}E_{\tau }^{\;n}(\{x\}),$$

hence there exists an integer n≥0 such that $y\in E_{\tau }^{\;n}(\{x\}).$

Now, let $z\in {\overset{―}{\left\{y\right\}}}_{g}^{\;\tau }$ be arbitrary. Again, by definition,

$${\overset{―}{\left\{y\right\}}}_{g}^{\;\tau }=\overset{\infty }{\underset{m=0}{⋃}}E_{\tau }^{\;m}(\{y\}),$$

so there exists an integer m≥0 such that $z\in E_{\tau }^{\;m}(\{y\}).$

We claim that $E_{\tau }^{\;m}(\{y\})\subseteq E_{\tau }^{\;n+m}(\{x\}).$ To justify this, note that the operator $E_{\tau }$ is monotone with respect to set inclusion. Since

$$\{y\}\subseteq E_{\tau }^{\;n}(\{x\}),$$

applying $E_{\tau }^{\;m}$ to both sides yields

$$E_{\tau }^{\;m}(\{y\})\subseteq E_{\tau }^{\;m}(E_{\tau }^{\;n}(\{x\}))=E_{\tau }^{\;n+m}(\{x\}).$$

Therefore,

$$z\in E_{\tau }^{\;n+m}(\{x\})\subseteq \overset{\infty }{\underset{k=0}{⋃}}E_{\tau }^{\;k}(\{x\})={\overset{―}{\left\{x\right\}}}_{g}^{\;\tau }.$$

Since z was arbitrary, it follows that

$${\overset{―}{\left\{y\right\}}}_{g}^{\;\tau }\subseteq {\overset{―}{\left\{x\right\}}}_{g}^{\;\tau }.$$

**Theorem 3.6.**

The relation ${∼}_{\tau }$ is an equivalence relation on X. Moreover, each equivalence class coincides with a maximal τ-connected granular substructure of A⊆X.

*Proof*. Reflexivity follows since $\rho_{\tau }(x,x)=0$. Symmetry is obvious by definition. For transitivity, assume $x{∼}_{\tau }y$ and $y{∼}_{\tau }z$. Then, by lemma 3.2, $z\in {\overset{―}{\left\{y\right\}}}_{g}^{\;\tau }\subseteq {\overset{―}{\left\{x\right\}}}_{g}^{\;\tau }$, hence $x{∼}_{\tau }z$.

Now, let $[a{]}_{{∼}_{\tau }}$ denote the equivalence class of a∈A. By definition,

$$[a{]}_{{∼}_{\tau }}=\{b\in A:a{∼}_{\tau }b\}.$$

For any $x,y\in [a{]}_{{∼}_{\tau }}$, we have $x{∼}_{\tau }a\;\text{and}\;a{∼}_{\tau }y.$ By transitivity, this implies $x{∼}_{\tau }y,$ so $[a{]}_{{∼}_{\tau }}$ is τ-connected.

To prove maximality, suppose that there exists a τ-connected set C⊆A such that $[a{]}_{{∼}_{\tau }}⊊C$, then there exists $z\in C\setminus [a{]}_{{∼}_{\tau }}$. Since C is τ-connected for any $x\in [a{]}_{{∼}_{\tau }}$, there exist τ-paths from x to z and from z to x. In particular, this holds for x=a, implying $a{∼}_{\tau }z,$ which contradicts $z\notin [a{]}_{{∼}_{\tau }}$. Therefore, $[a{]}_{{∼}_{\tau }}$ is maximal with respect to τ-connectivity.

Hence, each equivalence class of ${∼}_{\tau }$ coincides with a maximal τ-connected granular substructure of A⊆X.

**Theorem 3.7.**

Let (X,τ) be a soft topological space endowed with a proximity mapping τ, and let A⊆X. Let $\tau_{A}:A\to 2^{A}$ be the induced proximity mapping defined by $\tau_{A}(x)=\tau (x)\cap A.$ Let C⊆A be an equivalence class of the relation ${∼}_{\tau_{A}}$. Then, C is granularly saturated, that is, $E_{\tau_{A}}(C)=C.$

*Proof*. Let x∈C be arbitrary. By definition of equivalence class, for every y∈C, there exist $\tau_{A}$-paths from x to y and from y to x contained in A.

First, we show that $E_{\tau_{A}}(C)\subseteq C$. Let $z\in E_{\tau_{A}}(C)$. Then, there exists y∈C such that $z\in \tau_{A}(y)=\tau (y)\cap A.$ Hence, (y,z) is a $\tau_{A}$-path in A. By concatenating this path with an existing $\tau_{A}$-path from x to y, we obtain a $\tau_{A}$-path from x to z. Similarly, concatenating paths from z to y and from y to x yields a $\tau_{A}$-path from z to x. Therefore, $x{∼}_{\tau_{A}}z,$ which implies z∈C.

Next, since $x\in \tau_{A}(x)$ for all x∈A, we have $x\in E_{\tau_{A}}(\{x\})\subseteq E_{\tau_{A}}(C)$, and hence $C\subseteq E_{\tau_{A}}(C)$. Combining both inclusions, we conclude that $E_{\tau_{A}}(C)=C.$

**Theorem 3.8.**

Let (X,τ) be a soft topological space and let A⊆X. Let $\tau_{A}:A\to 2^{A}$ be the induced proximity mapping defined by $\tau_{A}(x)=\tau (x)\cap A.$ Let C⊆A be a soft granular community with respect to $\tau_{A}$. Then, the granular core ${\text{Core}}_{\tau_{A}}(C)$ is granularly saturated, that is, $E_{\tau_{A}}({\text{Core}}_{\tau_{A}}(C))={\text{Core}}_{\tau_{A}}(C).$

*Proof*. We have ${\text{Core}}_{\tau_{A}}(C)=\left\{x\in C|\tau_{A}(x)\subseteq C\right\}.$

First, we prove $E_{\tau_{A}}({\text{Core}}_{\tau_{A}}(C))\subseteq {\text{Core}}_{\tau_{A}}(C)$. Let $z\in E_{\tau_{A}}({\text{Core}}_{\tau_{A}}(C))$. By definition, there exists an element $y\in {\text{Core}}_{\tau_{A}}(C)$ such that $z\in \tau_{A}(y).$ Since $y\in {\text{Core}}_{\tau_{A}}(C)$, we have by definition that $\tau_{A}(y)\subseteq C.$ Hence, z∈C.

To show that $z\in {\text{Core}}_{\tau_{A}}(C)$, it remains to verify that $\tau_{A}(z)\subseteq C.$ Let $w\in \tau_{A}(z)$ be arbitrary. Since $z\in \tau_{A}(y)$ and $y\in {\text{Core}}_{\tau_{A}}(C)$ i.e. $\tau_{A}(y)\subseteq C$, it implies that z∈C. Since C is a soft granular community such that $w\in \tau_{A}(z)$ and z∈C, we have w∈C. Since w is arbitrary, it follows that $\tau_{A}(z)\subseteq C,$ which implies $z\in {\text{Core}}_{\tau_{A}}(C).$ Thus, $E_{\tau_{A}}({\text{Core}}_{\tau_{A}}(C))\subseteq {\text{Core}}_{\tau_{A}}(C).$

Now, we prove ${\text{Core}}_{\tau_{A}}(C)\subseteq E_{\tau_{A}}({\text{Core}}_{\tau_{A}}(C))$. Let $x\in {\text{Core}}_{\tau_{A}}(C)$. Since $\tau_{A}(x)$ is a neighbourhood of x, we have $x\in \tau_{A}(x).$ Hence, $x\in E_{\tau_{A}}(\{x\})\subseteq E_{\tau_{A}}({\text{Core}}_{\tau_{A}}(C)).$ Therefore, ${\text{Core}}_{\tau_{A}}(C)\subseteq E_{\tau_{A}}({\text{Core}}_{\tau_{A}}(C)).$

**Remark 3.3.**

Let (X,τ) be a soft topological space and C be a soft granular community. If ${\text{Bnd}}_{\tau }(C)\neq \emptyset$, then it is not granularly saturated.

*Proof*. For $x\in {\text{Bnd}}_{\tau }(C)$, there exists y∈τ(x)∩(X∖C). Hence, $E_{\tau }({\text{Bnd}}_{\tau }(C))$ intersects X∖C, so the equality $E_{\tau }({\text{Bnd}}_{\tau }(C))={\text{Bnd}}_{\tau }(C)$ cannot hold.

**Remark 3.4.**

Theorem 3.7 characterizes internal cohesion of a soft granular community through stability under the induced proximity mapping, whereas boundary effects with respect to the ambient topology (i.e. original soft proximity τ on X) reveal potential external influence. This dual perspective naturally reflects the multi-level granularity inherent in complex social networks, where communities may be internally stable yet externally exposed.

**Theorem 3.9.**

Let (X,τ) be a soft topological space. Then, for all A⊆X and k≤m, $I_{\tau }^{k}(A)\subseteq I_{\tau }^{m}(A).$

*Proof*. Let A⊆X and let k,m∈N with k≤m. We show that $I_{\tau }^{k}(A)\subseteq I_{\tau }^{m}(A).$ Let $y\in I_{\tau }^{k}(A)$. By the definition of $I_{\tau }^{k}(A)$, there exists x∈A such that $\rho_{\tau }(x,y)\leq k.$ Since k≤m, it follows immediately that $\rho_{\tau }(x,y)\leq m$. Again, by the definition of $I_{\tau }^{m}(A)$, we obtain $y\in I_{\tau }^{m}(A).$ Hence, every element of $I_{\tau }^{k}(A)$ belongs to $I_{\tau }^{m}(A)$, and therefore $I_{\tau }^{k}(A)\subseteq I_{\tau }^{m}(A)$. This completes the proof.

## 4. Applications to multi-criteria social network analysis

In this section, we demonstrate how the proposed soft topological granular framework can be employed to address fundamental analytical queries in multi-criteria social network analysis. Rather than attempting to model all possible network tasks, we focus on a small number of structurally significant questions that naturally arise in networks characterized by context-dependent interactions and multiple evaluation criteria.

Specifically, we concentrate on the following three queries:

(Q1) *Community formation under multiple criteria:* how can stable communities be identified when social ties are influenced by several contextual factors (such as trust [55], shared interests or interaction frequency [53,74]) without imposing sharp or disjoint boundaries?
(Q2) *Influence propagation and stabilization:* given an initial group of agents, how does influence spread through the network across different criteria, and under what conditions does this propagation stabilize into a persistent structure?
(Q3) *Identification of structurally influential agents:* which individuals act as core or anchor nodes whose local neighbourhoods remain invariant under the influence dynamics induced by multiple criteria?

The soft topological approach developed in this work is particularly well suited to these queries, as it models neighbourhoods through proximity mappings rather than fixed graph edges. This allows social relationships to be represented as overlapping and context-sensitive information granules. As will be shown, granular closure, reachability and saturation provide natural mathematical tools for community detection, influence analysis and core identification within a unified theoretical framework. Here, we also present two representative algorithms that encapsulate the dynamic and structural aspects of the proposed framework; additional procedures follow analogously from the established results.

### 4.1. Multi-criteria social network analysis via soft granular structure

We now formalize a social network structure based on the soft topological granular framework developed in the previous sections.

**Definition 4.1.**

A soft topological social network is a triple N=(X,C,τ), where

— X is a finite, non-empty set whose elements represent social agents (nodes) in the network,
— C is a finite set of social criteria (such as trust [55], communication frequency [75], shared interests [53], etc.), and
— $\tau :X\to 2^{X}$ is a proximity mapping induced by C, called the soft social proximity, satisfying x∈τ(x),∀x∈X.

The pair (X,τ) is called the soft topological social space associated with N.

**Remark 4.1.**

The proximity mapping τ is assumed to aggregate the effects of the criteria in C. For a given agent x∈X, the neighbourhood τ(x) represents the set of agents that are socially proximal to x under one or more criteria. Different modelling choices for τ correspond to different ways of combining criteria.

**Definition 4.2.**

For each criterion c∈C, let $\tau_{c}:X\to 2^{X}$ be a proximity mapping reflecting social closeness under criterion c. The aggregated social proximity τ may then be defined as

$$\tau (x)=\underset{c\in C}{⋃}\tau_{c}(x),\;x\in X.$$

**Remark 4.2.**

In the proposed structure, each neighbourhood τ(x) constitutes a local information granule centred at agent x. Overlapping neighbourhoods reflect agents sharing social contexts under multiple criteria. Higher-level granules emerge through reachability and granular closure, corresponding to communities and influence domains in the network.

Before formally stating the main results, we briefly explain the mechanism captured by the following theorem. Starting from an initial set of agents, the soft topological structure induces an iterative expansion through context-dependent neighbourhoods. This expansion aggregates agents who are indirectly connected via shared criteria to form an information granule that reflects collective social behaviour. The theorem below establishes that this process always converges to a well-defined, stable and minimal social community, independent of the order in which interactions occur.

**Theorem 4.1.**

Let N=(X,C,τ) be a soft topological social network and let A⊆X be a non-empty finite set of agents. Then, the granular closure ${\overset{―}{A}}_{g}^{\;\tau }$ is a τ-connected, τ-closed and granularly saturated subset of X. Moreover, ${\overset{―}{A}}_{g}^{\;\tau }$ is the smallest such subset containing A.

We provide a complete proof for clarity, as this result illustrates how the abstract soft granular topology directly governs social interaction structures.

*Proof*. Let A⊆X be non-empty. Define a sequence of subsets of X by

$$A_{0}=A,\;A_{k+1}=E_{\tau }(A_{k})\;(k\geq 0),$$

where $E_{\tau }(A_{k})=\underset{x\in A_{k}}{⋃}\tau (x)$.

Since x∈τ(x) for all x∈X, we have $A_{k}\subseteq A_{k+1},\forall k\geq 0.$ Hence, $\left\{A_{k}\right\}$ is an increasing sequence of subsets of X with respect to set inclusion. As X is finite, there exists a least integer $k_{0}$ such that $A_{k_{0}}=A_{k_{0}+1}.$ Hence, by definition of the operator E, we have, $E_{\tau }(A_{k_{0}})=A_{k_{0}}.$ From $E_{\tau }(A_{k_{0}})=A_{k_{0}}$, it follows that $E_{\tau }(A_{k_{0}})\subseteq A_{k_{0}},$ hence $A_{k_{0}}$ is τ-closed. Since $A_{k_{0}}\subseteq E_{\tau }(A_{k_{0}})$ always holds, $A_{k_{0}}$ is granularly saturated. Now, by definition of granular closure, ${\overset{―}{A}}_{g}^{\;\tau }=A_{k_{0}}.$ Let $x,y\in {\overset{―}{A}}_{g}^{\;\tau }$. Then, there exist integers m and n such that $x\in A_{m},y\in A_{n}.$ Without loss of generality, assume m≤n. Since $A_{m}\subseteq A_{n}$ and $A_{n+1}=E_{\tau }(A_{n})$, there exists a τ-path from x to y contained in ${\overset{―}{A}}_{g}^{\;\tau }$. Thus, every point is reachable from every other point, and ${\overset{―}{A}}_{g}^{\;\tau }$ is τ-connected. Now, let C be any τ-closed and τ-connected subset containing A. Since A⊆C and C is τ-closed, an induction on k yields $A_{k}\subseteq C$ for all k. Hence, ${\overset{―}{A}}_{g}^{\;\tau }\subseteq C.$ Therefore, ${\overset{―}{A}}_{g}^{\;\tau }$ is the smallest such subset.

This theorem provides a mathematically rigorous foundation for community formation in multi-criteria social networks. Starting from any initial group of agents, the granular closure ${\overset{―}{A}}_{g}^{\;\tau }$ captures all agents that become socially connected to the group through iterative neighbourhood expansion under multiple criteria. The resulting community is stable as no external agents can be absorbed, connected; i.e. information can flow internally and minimal.

**Theorem 4.2.**

Let N=(X,C,τ) be a soft topological social network and let A⊆X be a set of initially influenced agents. Define a sequence $\{A_{k}{\}}_{k\geq 0}$ by

$$A_{0}=A\;\text{and}\;A_{k+1}=E_{\tau }(A_{k}).$$

Then, there exists a least integer $k_{0}$ such that $A_{k}={\overset{―}{A}}_{g}^{\;\tau }\;\text{for all }k\geq k_{0}.$ That is, influence propagation stabilizes precisely at the granular closure of A.

*Proof*. Let N=(X,C,τ) be a soft topological social network, and $\{A_{k}{\}}_{k\in N}$ is a monotone increasing sequence by theorem 3.1, with respect to set inclusion.

Next, we show by induction that $A_{k}\subseteq {\overset{―}{A}}_{g}^{\;\tau }\;\text{for all }k.$ For k=0, this holds since $A\subseteq {\overset{―}{A}}_{g}^{\;\tau }$. Assume $A_{k}\subseteq {\overset{―}{A}}_{g}^{\;\tau }$. From proposition 3.1, since ${\overset{―}{A}}_{g}^{\;\tau }$ is τ-closed, hence $E_{\tau }({\overset{―}{A}}_{g}^{\;\tau })\subseteq {\overset{―}{A}}_{g}^{\;\tau }.$ By monotonicity of $E_{\tau }$, we have $E_{\tau }(A_{k})\subseteq E_{\tau }({\overset{―}{A}}_{g}^{\;\tau })\subseteq {\overset{―}{A}}_{g}^{\;\tau }$. Hence, $A_{k+1}\subseteq {\overset{―}{A}}_{g}^{\;\tau }$.

Now, since $\left\{A_{k}\right\}$ is increasing and bounded above by ${\overset{―}{A}}_{g}^{\;\tau }$, there exists a least $k_{0}\in N$ such that $A_{k_{0}}=A_{k_{0}+1}=E_{\tau }(A_{k_{0}})$. Then, $E_{\tau }(A_{k_{0}})\subseteq A_{k_{0}}$, so $A_{k_{0}}$ is τ-closed. Because $A_{k_{0}}$ is τ-closed and contains A, and ${\overset{―}{A}}_{g}^{\;\tau }$ is the smallest such set, so we have ${\overset{―}{A}}_{g}^{\;\tau }\subseteq A_{k_{0}}$. Again, since $A_{k}\subseteq {\overset{―}{A}}_{g}^{\;\tau }\;\text{for all }k,$ we must have $A_{k_{0}}={\overset{―}{A}}_{g}^{\;\tau }.$ Thus, by monotonicity, $A_{k}=A_{k_{0}}$ for all $k\geq k_{0}$.

This theorem provides a rigorous explanation for the stabilization of influence in multi-criteria social networks. Starting from an initial set of agents, repeated social interactions propagate influence through overlapping and context-dependent neighbourhoods. The result guarantees that this process always converges to a unique and stable region of influence, represented by the granular closure. Importantly, the stabilized influence region does not depend on the duration of propagation beyond a certain point, reflecting the emergence of persistent social norms, opinions or behaviours. This offers a mathematically grounded alternative to classical diffusion models based on fixed graph distances.

**Figure 3.**
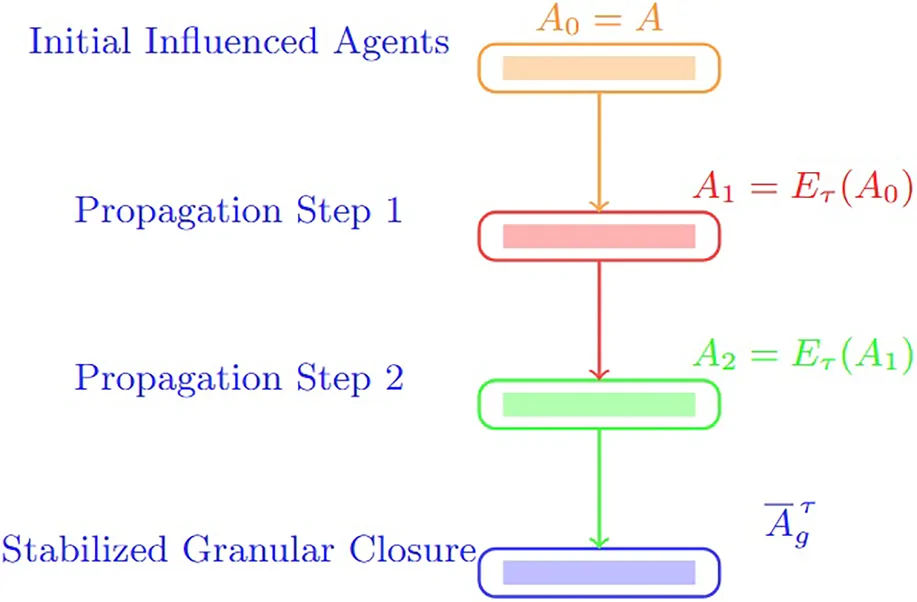
Visualization of influence propagation in a soft topological social network. Each layer corresponds to a propagation step, and the process stabilizes at the granular closure ${\overset{―}{A}}_{g}^{\;\tau }$.

Figure 3 visualizes the process of influence propagation in a soft topological social network through successive applications of τ-neighbourhood expansions. Each layer represents a propagation step, where influence originating from an initial set of agents spreads to their neighbouring agents according to the underlying soft topological structure. The layered arrangement highlights the dynamic and cumulative nature of information diffusion, with each successive stage incorporating agents that become reachable through local interactions. As the propagation continues, no new agents are added beyond a certain stage, and the process attains a stable configuration represented by the granular closure ${\overset{―}{A}}_{g}^{\;\tau }$. This closure comprises all agents that are eventually influenced under repeated neighbourhood expansion and characterizes the maximal region of influence generated by the initial set. Consequently, the figure provides an intuitive interpretation of how local interactions, when iteratively aggregated, give rise to a globally stabilized granular structure within the social network.

Algorithm 1 provides a formal description of influence propagation in a soft topological social network. Starting from an initial set of agents, the algorithm iteratively expands influence through soft neighbourhoods until a stable configuration is reached. By theorem 4.2, the output coincides with the granular closure of the initial set.

**Algorithm 1 Granular influence stabilization via soft expansion.**

**Figure:**
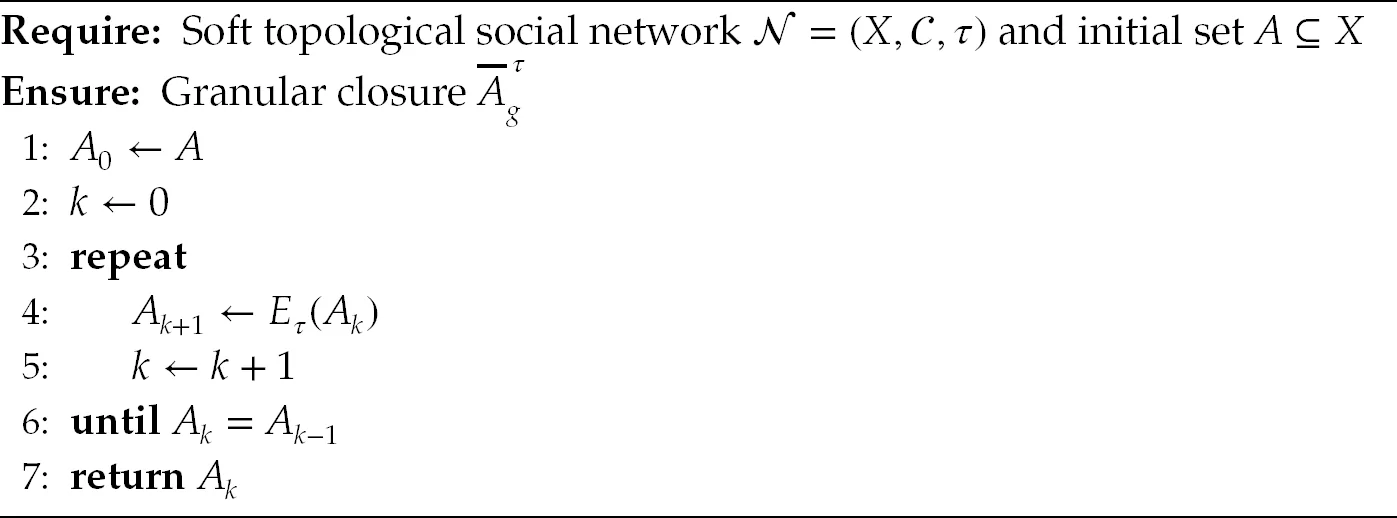

Algorithm 1 provides an operational realization of the theoretical results developed in section 3.2 and demonstrates how soft topological granulation can be applied to real-world social networks. In the following theorem, we assume that the soft topological social network N=(X,C,τ) is finite for real-world feasibility.

**Theorem 4.3.**

Let N=(X,C,τ) be a finite soft topological social network and let A⊆X. Then, algorithm 1 terminates after finitely many iterations and returns precisely the granular closure ${\overset{―}{A}}_{g}^{\;\tau }$ of A.

*Proof*. Define

$$A_{0}=A,\;A_{k+1}=E_{\tau }(A_{k}),\;k\geq 0.$$

Since the expansion operator is extensive by theorem 3.1, we have

$$A_{k}\subseteq A_{k+1}\;\text{for all }k\geq 0.$$

Hence, $A_{0}\subseteq A_{1}\subseteq A_{2}\subseteq \cdots$ is an increasing sequence of subsets of X. Since X is finite, there are only finitely many subsets of X. Therefore, there exists an integer m≥0 such that $A_{m}=A_{m+1}$. Consequently, the algorithm terminates.

By construction,

$$A_{m}=\underset{k\geq 0}{⋃}E_{\tau }^{\;k}(A),$$

which is precisely the definition of the granular closure ${\overset{―}{A}}_{g}^{\;\tau }$. Therefore, the output returned by algorithm 1 coincides with the granular closure of A.

We now address a finer analytical question concerning social networks: while granular closures describe the expansion of communities or influence, they do not distinguish agents that remain structurally stable under all admissible interaction criteria. In real social networks, such agents act as resilient cores whose local neighbourhoods do not extend beyond the community itself. The following result formalizes this intuition by characterizing the soft topological core of a granular community and proving its stability under neighbourhood expansion.

**Theorem 4.4.**

Let N=(X,C,τ) be a soft topological social network on a non-empty finite set X and let C⊆X be a non-empty soft granular community. Then, the soft granular core,

$${\text{Core}}_{\tau }(C)=\{x\in C:\tau (x)\subseteq C\},$$

is non-empty and granularly saturated.

*Proof*. Let C⊆X be a soft granular community. By lemma 3.1, there exists a subset A⊆X such that

$$C={\overset{―}{A}}_{g}^{\;\tau }=\overset{\infty }{\underset{n=0}{⋃}}E_{\tau }^{\;n}(A).$$

We claim that there exists x∈C such that τ(x)⊆C. Because C is τ-closed by definition of granular closure, we have $E_{\tau }(C)\subseteq C.$ Hence, for every x∈C, $\tau (x)\subseteq E_{\tau }(C)\subseteq C$. Therefore, ${\text{Core}}_{\tau }(C)\neq \emptyset .$

Next, let $x\in {\text{Core}}_{\tau }(C)$. By definition, for any y∈τ(x)⊆C, we have $\tau (y)\subseteq E_{\tau }(C)$. Since C is τ-closed, hence

$$\tau (y)\subseteq E_{\tau }(C)\subseteq C.$$

Thus, τ(y)⊆C, implying $y\in {\text{Core}}_{\tau }(C)$. Hence, $\tau (x)\subseteq {\text{Core}}_{\tau }(C),$ which shows that $E_{\tau }({\text{Core}}_{\tau }(C))\subseteq {\text{Core}}_{\tau }(C).$ On the other hand, since x∈τ(x) for all x∈X, we have the trivial inclusion ${\text{Core}}_{\tau }(C)\subseteq E_{\tau }({\text{Core}}_{\tau }(C)).$

Combining both inclusions, we conclude that $E_{\tau }({\text{Core}}_{\tau }(C))={\text{Core}}_{\tau }(C),$ and therefore ${\text{Core}}_{\tau }(C)$ is granularly saturated.

**Figure 4.**
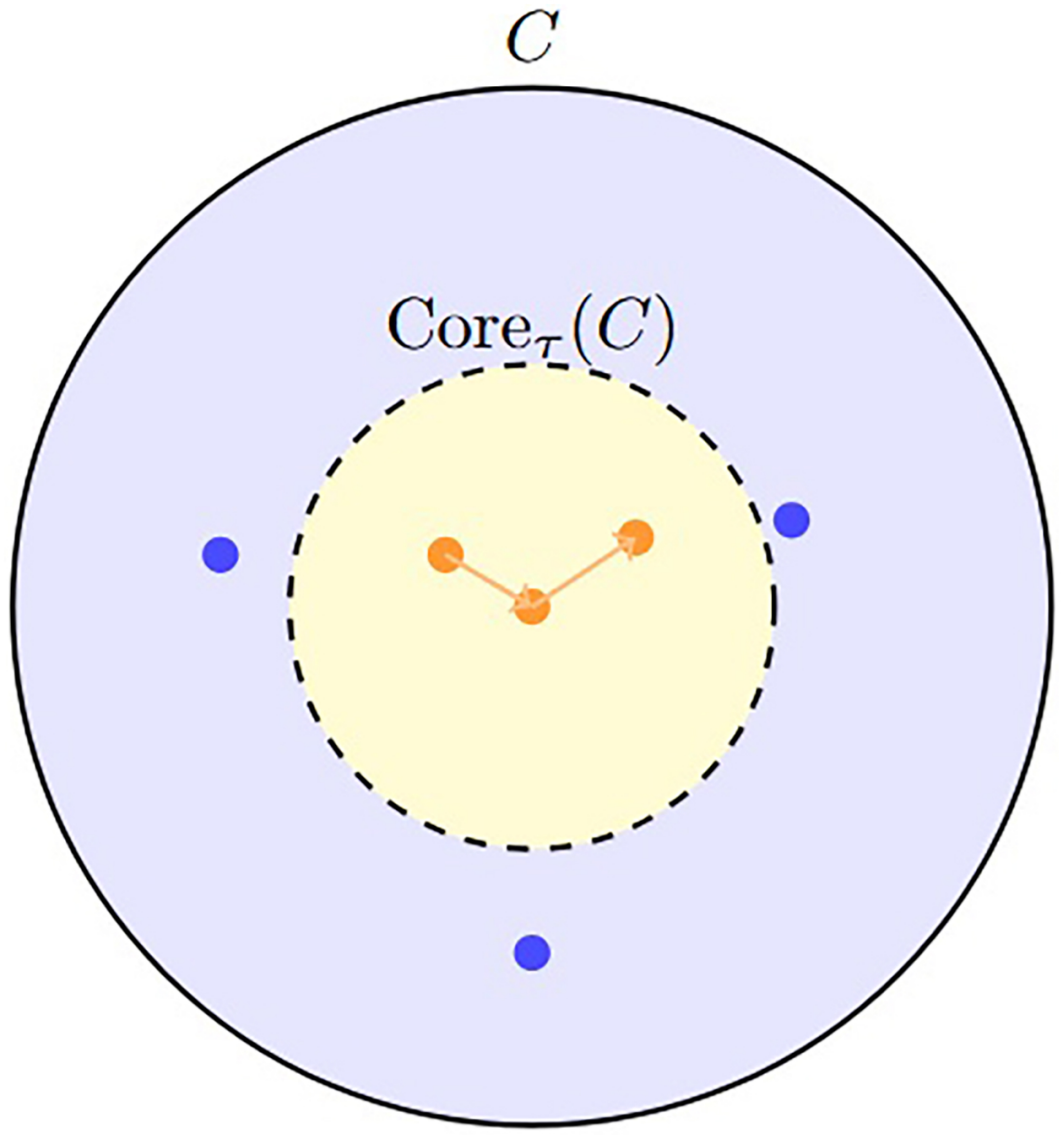
A granular community C with a stable core. Core nodes (orange) have neighbourhoods entirely contained within C, whereas peripheral nodes (blue) are part of the community but not in the core.

This theorem identifies a mathematically well-defined notion of stable core agents in a multi-criteria social network. Agents belonging to ${\text{Core}}_{\tau }(C)$ interact exclusively within their community under all admissible contexts, making them robust against external influence. Such nodes correspond to opinion leaders, ideological anchors or tightly bonded subgroups whose behaviour is invariant under local perturbations. Unlike degree-based or centrality-based cores, the present notion is context-sensitive and naturally accommodates heterogeneous interaction criteria. The scenario is pictorically shown in figure 4. It illustrates a granular community C together with its stable core structure in a soft topological social network. The orange nodes represent the core of the community, consisting of those agents whose τ-neighbourhoods are entirely contained within C. By contrast, the blue nodes are peripheral members of C. Although they belong to the community, their neighbourhoods are not completely contained within C, indicating potential interactions with agents outside the community. Thus, the figure highlights the distinction between the internally stable core and the more externally connected periphery, and provides a granular interpretation of community organization in soft topological social networks.

Algorithm 2 operationalizes the definition of the soft granular core by identifying agents whose τ-neighbourhoods are fully contained within the community.

**Algorithm 2 Extraction of the soft granular core of a community.**

**Figure:**
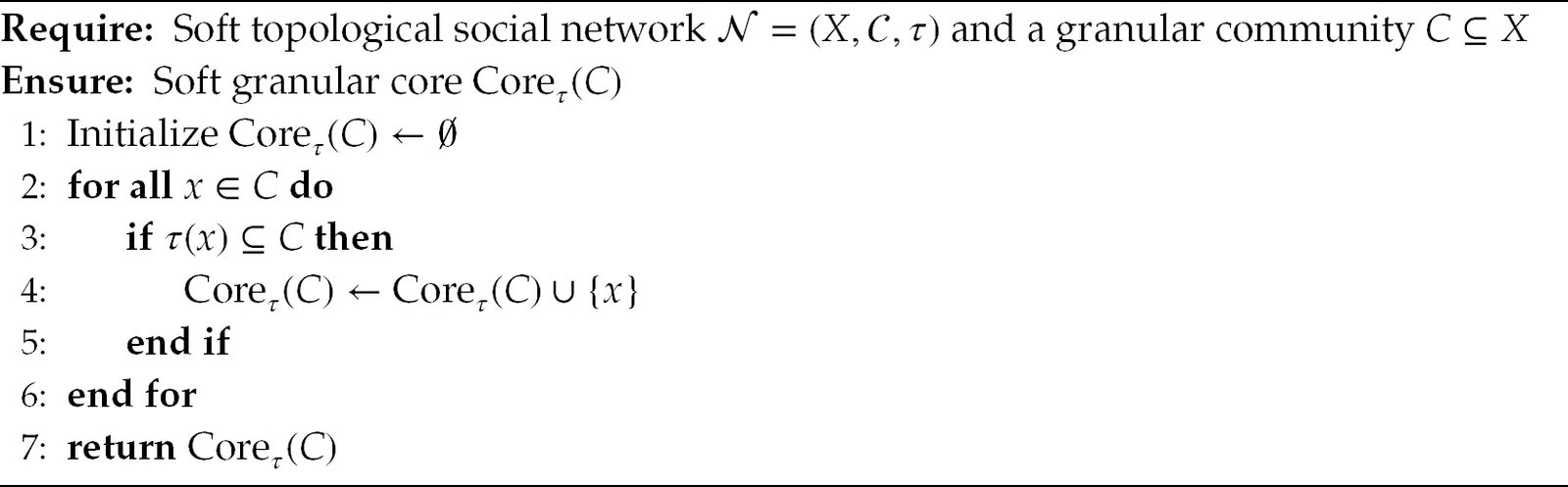

**Remark 4.3.**

Algorithm 2 is definitional in nature and serves to verify core membership rather than to compute an approximation. Its correctness follows directly from the definition of ${\text{Core}}_{\tau }(C)$ and the granular saturation of C.

However, we have the following intuitive theorem regarding the correctness of algorithm 2.

**Theorem 4.5.**

Let N=(X,C,τ) be a soft topological social network and let C⊆X be a granular community. Then, algorithm 2 returns exactly the soft granular core

$${\text{Core}}_{\tau }(C)=\{x\in C:\tau (x)\subseteq C\}.$$

*Proof*. For each element x∈C, the algorithm checks whether τ(x)⊆C. If this condition holds, then x is inserted into the output set. Hence, every element returned by the algorithm belongs to ${\text{Core}}_{\tau }(C)$.

Conversely, suppose $x\in {\text{Core}}_{\tau }(C)$. Then, by definition, τ(x)⊆C. Therefore, the condition in the algorithm is satisfied, and x is inserted into the output set. Thus, every element of ${\text{Core}}_{\tau }(C)$ is returned by the algorithm.

Hence, the output of algorithm 2 is precisely ${\text{Core}}_{\tau }(C)$.

**Theorem 4.6.**

Let (X,τ) be a soft topological space and GS(X,τ) be the associated soft topological granular structure. Then, the family of granular systems generated by (X,τ) is directed with respect to the pre-order ⪯; i.e for any two granular systems $G_{i}$ and $G_{j}$, there exists a granular system $G_{k}$ such that $G_{i}⪯G_{k}\;\text{and}\;G_{j}⪯G_{k}.$

*Proof*. Let $G_{i}$ and $G_{j}$ be two granular systems on X. Define $G_{k}=\{\text{Close}(G_{i}\cup G_{j},X,\tau ):\;G_{i}\in G_{i},\;G_{j}\in G_{j}\}.$ Each element of $G_{k}$ is a granule and hence belongs to the class of admissible granular systems generated by (X,τ).

Now, fix an arbitrary $G_{i}\in G_{i}$. Choose any $G_{j}\in G_{j}$. Then,

$$G_{i}\subseteq G_{i}\cup G_{j}\subseteq \text{Close}(G_{i}\cup G_{j},X,\tau ).$$

Thus, there exists $H\in G_{k}$ such that $G_{i}\subseteq H$, and therefore $G_{i}⪯G_{k}$. Similarly, the same argument shows that $G_{j}⪯G_{k}$.

Hence, the family of granular systems generated by (X,τ) is directed with respect to ⪯.

**Figure 5.**
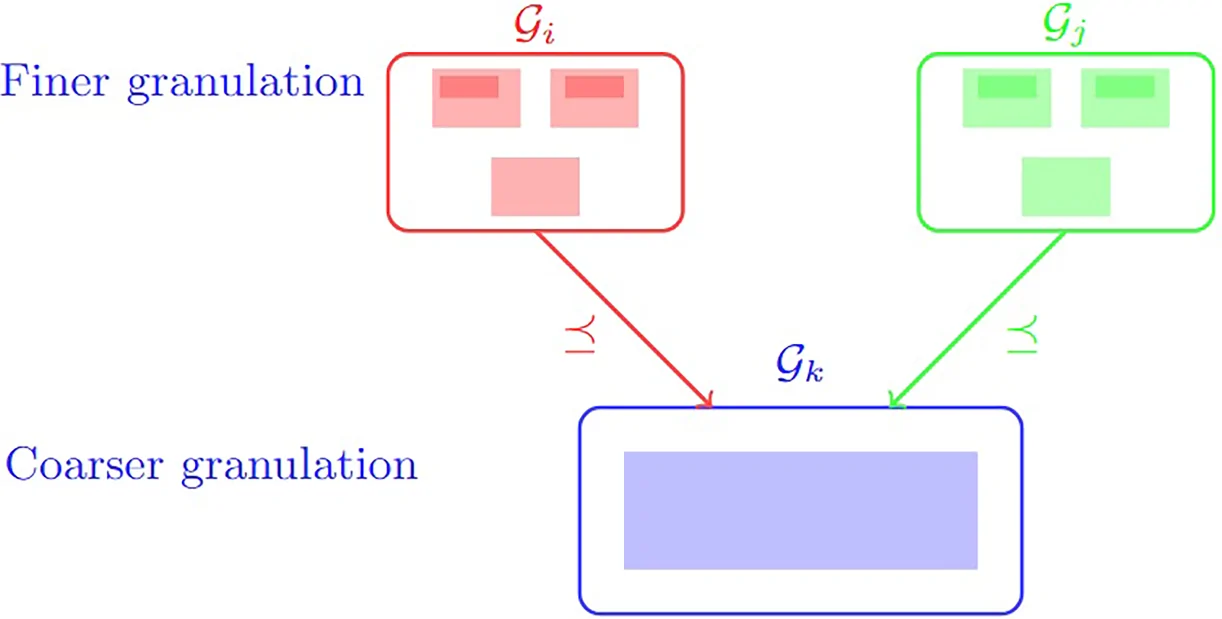
Directed structure of soft topological granular systems with an additional finer layer. Distinct granular systems $G_{i}$ and $G_{j}$ admit a common upper granulation $G_{k}$ with respect to the pre-order ⪯, reflecting hierarchical refinement and multi-resolution consistency.

The above theorem confirms that the proposed soft topological granular structure supports multi-level granulation in a rigorous sense. Any two granular descriptions derived from different interaction criteria or contextual viewpoints admit a common refinement. This property is fundamental in GrC, as it guarantees the consistency of reasoning across multiple abstraction levels and enables adaptive granularity selection in applications such as multi-criteria social network analysis. To illustrate the hierarchical and directed structure of soft topological granular systems, we depict in figure 5 the finer and coarser granules along with the pre-order relations among them. It illustrates the directed organization of soft topological granular systems under the pre-order relation ⪯. Each granular system, denoted by $G_{i}$, $G_{j}$ and $G_{k}$, represents a particular granulation of the underlying universe, corresponding to different levels of information resolution or neighbourhood refinement. The directed edges indicate the pre-order relation among these systems, where one granulation is regarded as finer or more informative than another. In particular, the figure depicts the existence of a common upper granulation $G_{k}$ that simultaneously refines the granular systems $G_{i}$ and $G_{j}$. This additional finer layer integrates the information contained in the lower granulations while preserving their structural relationships. Consequently, the diagram highlights the hierarchical nature of soft topological granular systems and demonstrates how multiple granular representations can be coherently organized within a unified framework. Such an arrangement provides a natural mechanism for multi-resolution analysis, and it allows information to be examined consistently across different levels of granularity while maintaining compatibility among distinct granular viewpoints.

### 4.2. Illustrative examples for the proposed soft topological granular computing framework

In order to illustrate the practical significance of the proposed soft topological granular framework, we present synthetic proximity-based network examples in which all of the fundamental notions introduced in this paper arise simultaneously. The example is deliberately constructed so that the induced soft topological space possesses multiple non-trivial soft granular communities, a non-trivial granular closure generated from a singleton set, and a subset having a non-empty granular core, boundary and periphery. Consequently, the examples provide a unified setting for visualizing the processes of information propagation, community formation and the internal and external structural organization of granular regions within a soft topological environment.

**Example 4.1.**

Let $X=\{x_{1},x_{2},\ldots ,x_{12}\}$ and define the proximity mapping $\tau :X\to 2^{X}$ by $\tau (x_{1})=\{x_{1},x_{2},x_{3}\},$$\tau (x_{2})=\{x_{1},x_{2},x_{4}\},$$\tau (x_{3})=\{x_{1},x_{3},x_{5}\},$$\tau (x_{4})=\{x_{2},x_{4},x_{5}\},$$\tau (x_{5})=\{x_{3},x_{4},x_{5}\},$$\tau (x_{6})=\{x_{6}\},\tau (x_{7})=$$\{x_{7},x_{8},x_{9}\},$$\tau (x_{8})=\{x_{7},x_{8},x_{10}\},$$\tau (x_{9})=\{x_{7},x_{9},x_{11}\},$$\tau (x_{10})=\{x_{8},x_{10},x_{12}\},$$\tau (x_{11})=\{x_{9},x_{11}\}\;\text{and}\;\tau (x_{12})=\{x_{10},x_{12}\}$.

First, consider $C_{1}=\{x_{1},x_{2},x_{3},x_{4},x_{5}\}$. Observe that every vertex of $C_{1}$ is mutually reachable from every other vertex of $C_{1}$. For example, $x_{1}\to x_{3}\to x_{5}$ implies that $\rho_{\tau }(x_{1},x_{5})<\infty$, while $x_{5}\to x_{3}\to x_{1}$ gives $\rho_{\tau }(x_{5},x_{1})<\infty$. Proceeding similarly, we obtain $x{∼}_{\tau }y\;\text{for all }x,y\in C_{1}$. Hence, $C_{1}=\{x_{1},x_{2},x_{3},x_{4},x_{5}\}$ is a soft granular community.

Similarly, $C_{2}=\{x_{7},x_{8},x_{9},x_{10},x_{11},x_{12}\}$ also forms a soft granular community since every pair of its vertices is mutually reachable. Similarly, $C_{3}=\{x_{6}\}$ is trivially a soft granular community. Moreover, $\rho_{\tau }(x_{1},x_{7})=\infty \;\text{,}\;\rho_{\tau }(x_{7},x_{1})=\infty \;\text{,}\;\rho_{\tau }(x_{1},x_{6})=\infty \;\text{and}\;\rho_{\tau }(x_{7},x_{6})=\infty$. Therefore, the soft granular communities are $C_{1}=\{x_{1},x_{2},x_{3},x_{4},x_{5}\}$, $C_{2}=\{x_{7},x_{8},x_{9},x_{10},x_{11},x_{12}\}$ and $C_{3}=\{x_{6}\}$.

Next, consider $A=\{x_{11}\}$. Then, $E_{\tau }(A)=\{x_{9},x_{11}\}$ and $E_{\tau }^{2}(A)=\{x_{7},x_{9},x_{11}\}$. Further, $E_{\tau }^{3}(A)=\{x_{7},x_{8},x_{9},x_{11}\}$ and $E_{\tau }^{4}(A)=\{x_{7},x_{8},x_{9},x_{10},x_{11}\}$. Finally, $E_{\tau }^{5}(A)=\{x_{7},x_{8},x_{9},x_{10},x_{11},x_{12}\}$. Since $E_{\tau }(\{x_{7},x_{8},x_{9},x_{10},x_{11},x_{12}\})=\{x_{7},x_{8},x_{9},x_{10},x_{11},x_{12}\}$, we obtain ${\overset{―}{\left\{x_{11}\right\}}}_{g}^{\tau }=\{x_{7},x_{8},x_{9},x_{10},x_{11},x_{12}\}=C_{2}$.

**Figure 6.**
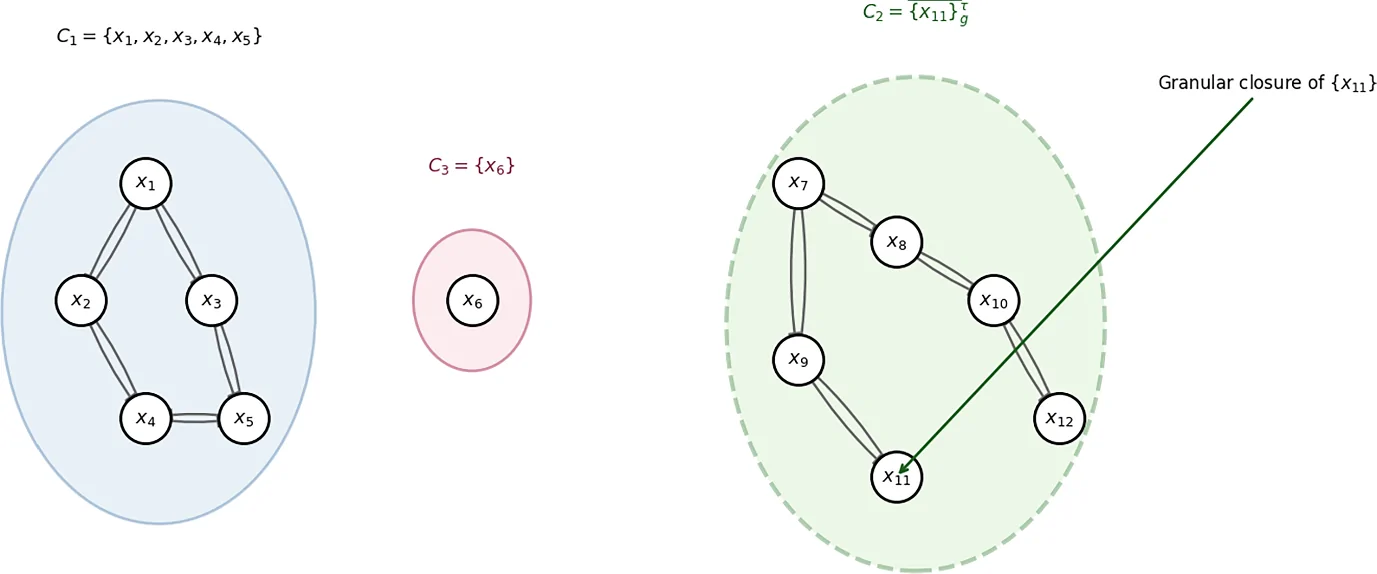
Soft granular communities induced by the proximity mapping τ. The shaded regions represent the three soft granular communities $C_{1}=\{x_{1},x_{2},x_{3},x_{4},x_{5}\}$, $C_{2}=\{x_{7},x_{8},x_{9},x_{10},x_{11},x_{12}\}$ and $C_{3}=\{x_{6}\}$. The dashed green region highlights that the community $C_{2}$ is also the granular closure ${\overset{―}{\left\{x_{11}\right\}}}_{g}^{\tau }$, illustrating how repeated neighbourhood aggregation starting from the singleton set $\left\{x_{11}\right\}$ stabilizes into a saturated information granule.

Figure 6 illustrates the soft topological granular structure associated with the proximity mapping τ. The vertices are partitioned into three soft granular communities, namely $C_{1}=\{x_{1},x_{2},x_{3},x_{4},x_{5}\}$, $C_{2}=\{x_{7},x_{8},x_{9},x_{10},x_{11},x_{12}\}$ and the isolated community $C_{3}=\{x_{6}\}$. The shaded regions represent coherent groups of vertices whose members are mutually reachable through repeated neighbourhood aggregation. In particular, the dashed green region corresponds to the community $C_{2}$, which is also the granular closure ${\overset{―}{\left\{x_{11}\right\}}}_{g}^{\tau }$. Starting from the singleton set $\left\{x_{11}\right\}$, successive applications of the aggregation operator $E_{\tau }$ progressively enlarge the information granule until it stabilizes at the saturated set $C_{2}$. The figure therefore provides a unified visualization of local neighbourhoods, multi-step information propagation, community formation and stabilization into a granular closure within the same soft topological framework.

**Example 4.2.**

Let $X=\{x_{1},x_{2},x_{3},x_{4},x_{5},x_{6},x_{7},x_{8}\}$ and define the proximity mapping $\tau :X\to 2^{X}$ by $\tau (x_{1})=\{x_{1},x_{2},x_{3}\},$$\tau (x_{2})=\{x_{1},x_{2},x_{4}\},$$\tau (x_{3})=\{x_{1},x_{3},x_{4}\},$$\tau (x_{4})=\{x_{2},x_{3},x_{4},x_{5},x_{6}\},$$\tau (x_{5})=$$\{x_{4},x_{5},x_{6}\},$$\tau (x_{6})=\{x_{4},x_{5},x_{6},x_{7}\},$$\tau (x_{7})=\{x_{6},x_{7},x_{8}\}\;\text{and}$$\tau (x_{8})=\{x_{7},x_{8}\}$.

Consider the subset $C=\{x_{1},x_{2},x_{3},x_{4}\}$. Then,

$$\begin{array}{rl} E_{\tau }(C) & =\tau (x_{1})\cup \tau (x_{2})\cup \tau (x_{3})\cup \tau (x_{4})\\ & =\{x_{1},x_{2},x_{3}\}\cup \{x_{1},x_{2},x_{4}\}\cup \{x_{1},x_{3},x_{4}\}\cup \{x_{2},x_{3},x_{4},x_{5},x_{6}\}\\ & =\{x_{1},x_{2},x_{3},x_{4},x_{5},x_{6}\}. \end{array}$$

Hence, ${\text{Per}}_{\tau }(C)=E_{\tau }(C)\setminus C=\{x_{5},x_{6}\}$. Next, observe that $\tau (x_{1})=\{x_{1},x_{2},x_{3}\}\subseteq C$, $\tau (x_{2})=\{x_{1},x_{2},x_{4}\}\subseteq C$ and $\tau (x_{3})=\{x_{1},x_{3},x_{4}\}\subseteq C$. Therefore, $x_{1},x_{2},x_{3}\in {\text{Core}}_{\tau }(C)$. Consequently, ${\text{Core}}_{\tau }(C)=\{x_{1},x_{2},x_{3}\}$. Now, $x_{4}\in C$ and $\tau (x_{4})\cap (X\setminus C)=\{x_{2},x_{3},x_{4},x_{5},x_{6}\}\cap \{x_{5},x_{6},x_{7},x_{8}\}\neq \emptyset$. Thus, ${\text{Bnd}}_{\tau }(C)=\{x_{4}\}$.

Therefore, the subset $C=\{x_{1},x_{2},x_{3},x_{4}\}$ possesses a non-empty granular core, a non-empty granular boundary and a non-empty granular periphery, thereby illustrating the internal, transitional and external regions of a granular structure in a soft topological environment.

**Figure 7.**
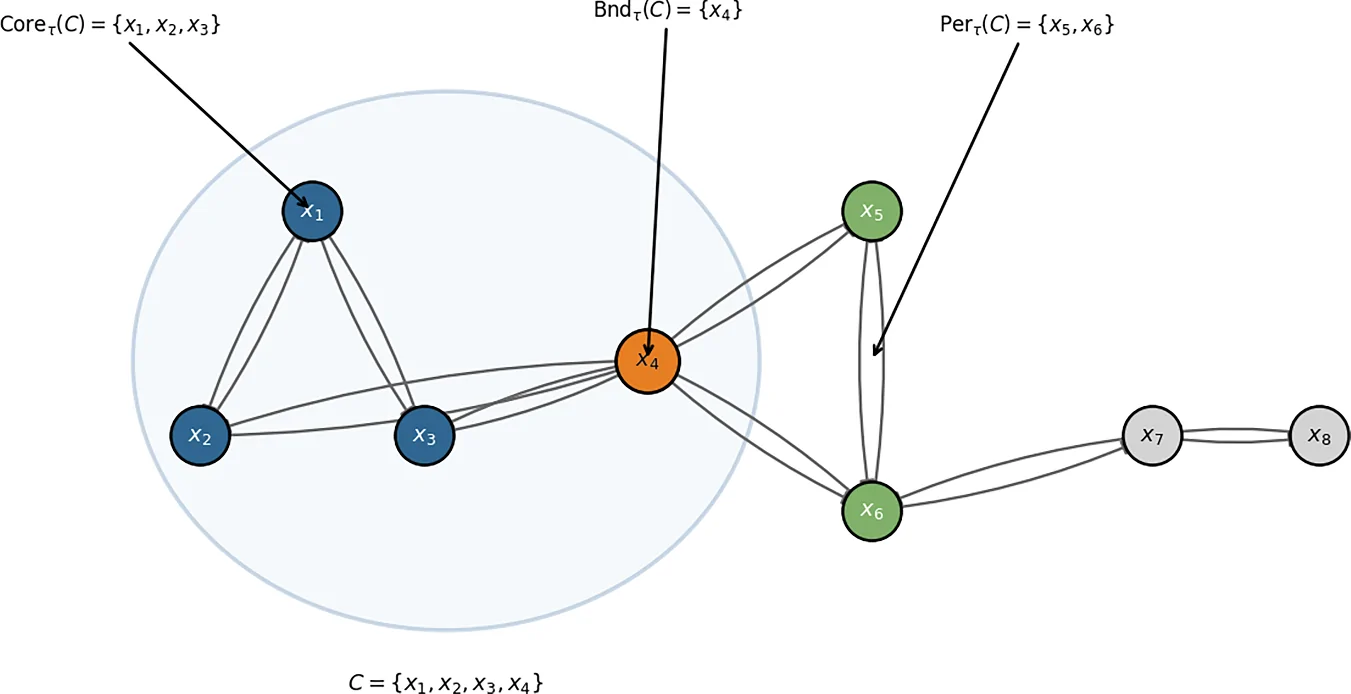
Decomposition of the subset $C=\{x_{1},x_{2},x_{3},x_{4}\}$ in a soft topological granular structure. The dark blue vertices form the granular core ${\text{Core}}_{\tau }(C)=\{x_{1},x_{2},x_{3}\}$, the orange vertex represents the granular boundary ${\text{Bnd}}_{\tau }(C)=\{x_{4}\}$ and the green vertices constitute the granular periphery ${\text{Per}}_{\tau }(C)=\{x_{5},x_{6}\}$. The figure illustrates the internal, transitional and external regions of a granular structure generated by the proximity mapping τ.

Figure 7 illustrates the decomposition of the subset $C=\{x_{1},x_{2},x_{3},x_{4}\}$ in the soft topological structure induced by the proximity mapping τ. The shaded region represents the set C, while the vertices are partitioned according to their structural roles within and around this subset. The dark blue vertices $x_{1},x_{2}$ and $x_{3}$ form the granular core ${\text{Core}}_{\tau }(C)$ since their neighbourhoods are entirely contained in C. The orange vertex $x_{4}$ constitutes the granular boundary ${\text{Bnd}}_{\tau }(C)$ because its neighbourhood intersects both C and its complement, thereby serving as a transitional vertex between the internal and external regions of the structure. The green vertices $x_{5}$ and $x_{6}$ form the granular periphery ${\text{Per}}_{\tau }(C)$, which consists of vertices that are generated through neighbourhood aggregation but do not belong to C. The remaining vertices $x_{7}$ and $x_{8}$ lie outside both C and its periphery. Thus, the figure provides a visual representation of how a soft topological granular structure naturally decomposes a subset into its internal, transitional and external components.

These examples highlight some useful features of the proposed soft topological granular framework when compared with traditional methods in network science. Classical diffusion models mainly study how information spreads through the edges of a network and often require additional assumptions, such as transmission probabilities or specific dynamic rules. By contrast, the operator $E_{\tau }$ and its iterates describe the propagation of influence directly from the given soft neighbourhood structure. They not only show how information spreads but also identify the final stable region reached by the propagation through the notion of granular closure. Similarly, many community detection algorithms find communities by optimizing certain criteria, such as modularity or network density, and treat communities as objects computed by an external procedure. In our framework, communities emerge naturally from the mutual reachability relation and therefore have a clear mathematical meaning. Moreover, traditional centrality measures assign numerical scores to indicate the importance of vertices, whereas the notions of granular core, boundary and periphery provide a more structural description by distinguishing internal, transitional and external parts of the network. Thus, the soft topological granular framework brings together information propagation, community formation and structural analysis within a single mathematical setting, while requiring fewer additional assumptions and remaining flexible enough to model complex and context-dependent interactions.

## 5. Discussion

This paper develops a soft topological framework for GrC by systematically integrating soft set-based neighbourhood structures with topological notions of closure, connectivity and convergence [43]. The primary objective was not merely to introduce new definitions, but to construct a mathematically coherent granular structure capable of modelling context-dependent and multi-criteria information systems, with particular emphasis on social network analysis [2].

The proposed soft topological granular structure unifies several foundational concepts in GrC [46]. Granules are generated through soft neighbourhoods rather than crisp equivalence classes [45], which allow them to reflect graded, parameter-dependent relationships. The introduction of granular expansion, closure, reachability and connectivity operators provides a dynamic view of information granulation, where granules evolve through influence propagation and stabilize as τ-closed sets. This perspective extends classical rough and fuzzy granular models [37] by embedding them within a topological process, and thus offers a principled mechanism for handling uncertainty, heterogeneity and interaction dynamics.

A key theoretical contribution of this work is the establishment of equivalence relations induced by granular reachability and the characterization of granular communities as saturated and τ-closed sets. These results show that stable information granules arise naturally as fixed points of soft topological operators, providing a strong mathematical justification for community formation and persistence in complex networks. Unlike purely combinatorial or graph-theoretic approaches [2], the proposed framework captures both local interactions and global structural properties through topological closure.

From the standpoint of GrC, the partial order relation among granular systems introduces a formal mechanism for comparing different levels of granularity. This allows one to study refinement and coarsening processes in a rigorous manner, which is essential for hierarchical analysis [39], multi resolution modelling [62] and adaptive information processing [44]. Such comparisons are particularly valuable in real-world applications where data representations evolve over time or across different parameter sets.

Table 3 summarizes the conceptual distinctions between the proposed soft topological granular framework and existing GrC [38,40,41] and social network analysis approaches [76–78]. Classical rough set and fuzzy granular models rely on fixed equivalence relations or quantitative membership functions, which impose structural or numerical assumptions on uncertainty representation. Graph-based social network models, in contrast, depend on predefined adjacency relations and often address multi-criteria interactions through heuristic aggregation. The proposed framework departs from these paradigms by deriving granules from soft topological neighbourhoods induced by parameter sets, allowing uncertainty to be captured intrinsically through context dependence rather than numerical approximation. Granules emerge dynamically via granular expansion and stabilize as τ-closed and saturated sets. It provides a mathematically guaranteed notion of community persistence and influence stabilization. Furthermore, the identification of granular cores through neighbourhood invariance offers a structural alternative to classical centrality measures. This comparison clarifies that the primary contribution of the present work lies in establishing a foundational theory that unifies granulation, stability and multi-criteria interaction within a single soft topological structure.

**Table 3.** Comparison of GrC and social network analysis frameworks.

aspect	classical rough sets	fuzzy/probabilistic granules	graph-based social networks	proposed soft topological granular framework
underlying foundation	equivalence relations	membership or probability measures	fixed graph topology	soft set induced topological structure
treatment of uncertainty	discrete indiscernibility	quantified vagueness	usually implicit	parameter-dependent, non-quantified uncertainty
neighbourhood structure	crisp equivalence classes	metric or similarity-based neighbourhoods	fixed adjacency relations	soft neighbourhood via proximity mappings
boundary of granules	sharp	gradual but predefined	sharp node sets	context-sensitive and overlapping boundaries
granule formation	static	mostly static	static or time-stepped	dynamic via granular expansion and closure
community stability	not intrinsic	measure-dependent	heuristic-dependent	guaranteed by τ-closure and saturation
multi-criteria handling	limited	aggregated numerically	often heuristic	intrinsic via soft parameter aggregation
core/influential agents	not explicitly defined	centrality-based notions	degree or betweenness measures	topologically invariant granular core
dependence on data or simulation	low	high	high	purely foundational and structural

The application to multi-criteria social network analysis illustrates the practical relevance of the theoretical framework. By modelling social interactions through soft neighbourhoods associated with multiple attributes (such as trust [55], frequency of interaction [74], shared interests [53], etc.), the proposed approach enables the identification of influence cores, stable communities and reachability-based information flow patterns.

Overall, the framework presented in this paper provides a flexible and mathematically robust foundation for advancing GrC in complex, uncertain and multi-criteria environments.

## 6. Conclusion

In this paper, we developed a soft topological framework for GrC that provides a rigorous mathematical foundation for representing context-dependent and multi-criteria information systems. By integrating soft set theory with topological concepts such as neighbourhood systems, closure operators, reachability, connectivity and convergence, we established a unified granular structure capable of describing both local interactions and global organizational properties. The proposed framework demonstrates that meaningful information granules naturally emerge as τ-closed and saturated sets and that these granules can be interpreted as stable communities and influence structures in multi-criteria social networks.

The theoretical development reveals that soft topological granules possess rich structural characteristics, including hierarchical organization, propagation behaviour and multi-resolution representations. The illustrative examples indicate that the framework can identify granular communities, influence cores and different levels of granularity while preserving the inherent uncertainty and contextual dependence of social interactions. Consequently, the proposed approach extends existing GrC paradigms by introducing a dynamic and topologically grounded perspective for analysing complex systems under uncertainty.

Several promising directions for future research arise from the present work. Potential extensions include the incorporation of weighted or temporal soft topologies, the study of granular dynamics under evolving parameter sets and the integration of the proposed framework with learning-based or probabilistic models. From a theoretical perspective, further investigation into categorical properties, homological invariants or algebraic structures associated with soft topological granules may yield deeper insights. On the other hand, one natural extension is the development of analogous granular structures in hypertopological [79] and superhypertopological settings [80], where granules themselves may be regarded as higher-order topological objects. Such investigations may provide deeper insights into hierarchical, higher-order and evolving network structures. Another important direction is the incorporation of richer uncertainty models through extensions based on fuzzy soft sets [16], neutrosophic soft sets [70] and plithogenic soft sets [81]. These generalizations would permit the simultaneous representation of vagueness, indeterminacy, inconsistency and multi-valued attribute interactions, and thus substantially broadening the applicability of soft topological GrC to decision-making, knowledge discovery, social network dynamics and adaptive intelligent systems.

## Data Availability

This study is purely theoretical. No datasets, code or external digital research materials were generated or analysed during the preparation of this manuscript. All results are derived analytically and are fully contained within the article.
